# The Kavaratamides: Discovery of Linear Lipodepsipeptides from the Marine Cyanobacterium *Moorena bouillonii* Using a Comparative Chemogeographic Analysis

**DOI:** 10.1021/acs.jnatprod.4c00242

**Published:** 2024-06-04

**Authors:** Byeol Ryu, Evgenia Glukhov, Thaiz R. Teixeira, Conor R. Caffrey, Saranya Madiyan, Valsamma Joseph, Nicole E. Avalon, Christopher A. Leber, C. Benjamin Naman, William H. Gerwick

**Affiliations:** † Center for Marine Biotechnology and Biomedicine, Scripps Institution of Oceanography, University of California San Diego, 9500 Gilman Drive, La Jolla, California 92093, United States; ‡ Center for Discovery and Innovation in Parasitic Diseases, Skaggs School of Pharmacy and Pharmaceutical Sciences, University of California San Diego, 9500 Gilman Drive, La Jolla, California 92093, United States; § National Centre for Aquatic Animal Health, Cochin University of Science and Technology, Kochi, Kerala 682016, India; ⊥ Department of Science and Conservation, San Diego Botanic Garden, 300 Quail Gardens Drive, Encinitas, California 92024, United States; ∥ Skaggs School of Pharmacy and Pharmaceutical Sciences, University of California San Diego, 9500 Gilman Drive, La Jolla, California 92093, United States

## Abstract

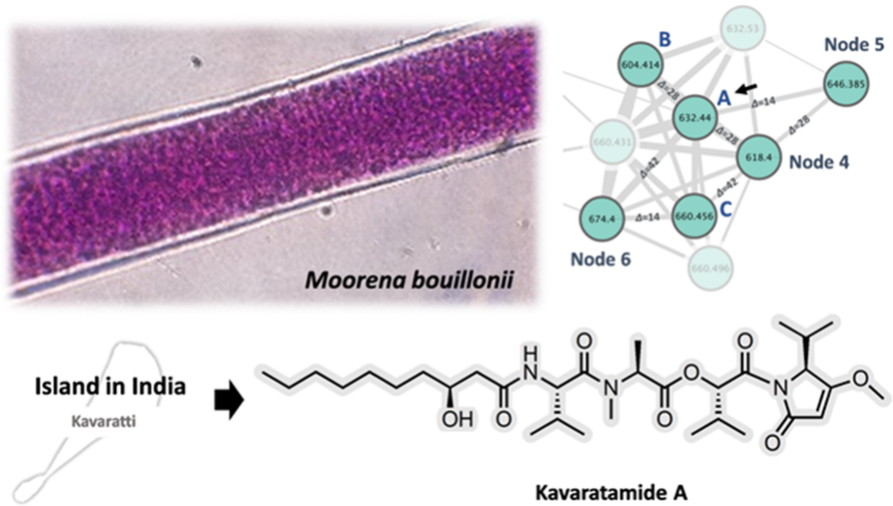

Kavaratamide A (**1**), a new linear lipodepsipeptide possessing an unusual isopropyl-*O*-methylpyrrolinone moiety, was discovered from the tropical marine filamentous cyanobacterium *Moorena bouillonii* collected from Kavaratti, India. A comparative chemogeographic analysis of *M*. *bouillonii* collected from six different geographical regions led to the prioritized isolation of this metabolite from India as distinctive among our data sets. AI-based structure annotation tools, including SMART 2.1 and DeepSAT, accelerated the structure elucidation by providing useful structural clues, and the full planar structure was elucidated based on comprehensive HRMS, MS/MS fragmentation, and NMR data interpretation. Subsequently, the absolute configuration of **1** was determined using advanced Marfey’s analysis, modified Mosher’s ester derivatization, and chiral-phase HPLC. The structures of kavaratamides B (**2**) and C (**3**) are proposed based on a detailed analysis of their MS/MS fragmentations. The biological activity of kavaratamide A was also investigated and found to show moderate cytotoxicity to the D283-medullablastoma cell line.

The tropical marine cyanobacterial genus *Moorena*, formerly classified as *Lyngbya* and subsequently as *Moorea*, is well established as a prolific source of biologically active secondary metabolites.^1^ For example, nearly 70 metabolites have been reported from the species *M*. *bouillonii* and include such notable compounds as the potent cytotoxin apratoxin A and its analogues,^2−9^ the lyngbyabellins,^10−15^ the columbamides,^16−18^ and the ulongamides.^19^ These previously reported compounds were identified in *M*. *bouillonii* collected from various locations, such as Guam, Palmyra Atoll, Malaysia, and Papua New Guinea. Because different environmental conditions and ecological factors in diverse habitats can contribute to the biosynthesis of distinct secondary metabolites, the presence of unique compounds in different habitats from the same organism is a common occurrence. Alternatively, geographically distinct areas can harbor chemically distinct genetic strains of a species. In either case, exploring organisms from regions that have not been previously studied increases the potential for uncovering new and unique natural products.^20^ Thus, a comparative analysis of organisms collected from multiple locations can allow the targeted isolation and identification of metabolites that are distinctive to a specific region. The application of Global Natural Products Social (GNPS) molecular networking using LC-MS/MS data can streamline this prioritization process.^21^ This tool evaluates the similarity between metabolites through their MS/MS fragmentations and then allows visualization of the molecular network. This dramatically reduces the labor-intensive process of manually comparing these spectra, thereby allowing a comparison of large numbers of samples and, in this case, identification of natural products distinctive to a particular geographical region. Previously, we reported on the isolation of several new natural products, the doscadenamides, obtained from *M*. *bouillonii* collected from Saipan.^20^ These were prioritized for isolation by applying a newly developed tool, namely, Objective Relational Comparative Analysis (ORCA) that is based on MS/MS molecular networking data. This ORCA approach compared the metabolite signature of 15 *M*. *bouillonii* samples collected from six different geographical regions, including India, China, Saipan, American Samoa, Guam, and Papua New Guinea.^20^

In the current study, the cyanobacterium *M*. *bouillonii* was collected from Kavaratti, India, which had previously been identified as possessing a unique metabolomic profile,^20^ and therefore was prioritized for a more detailed analysis. A combination of GNPS molecular networking and manual inspection of the LC-MS and LC-DAD traces identified a candidate new natural product from this sample. Here we describe the targeted isolation and structure elucidation of kavaratamide A (**1**), which features an uncommon PKS/NRPS-derived isopropyl-*O*-methylpyrrolinone (iPr-*O*-Me-pyr) moiety (Figure 1A). The planar structure of kavaratamide A (**1**) was elucidated through a combination of NMR and MS analyses, aided by AI-based structure annotation tools including SMART 2.1 and DeepSAT.^22,23^ The assignment of absolute configuration at all stereocenters was accomplished through a combination of techniques, including chemical degradation, advanced Marfey’s analysis, modified Mosher ester analysis, and chiral-phase HPLC. Also, the structures of two minor analogues, kavaratamides B (**2**) and C (**3**), are proposed based on a detailed analysis of their MS/MS fragmentation patterns and biosynthetic logic. The biological activity of kavaratamide A was explored in several assays and found to have moderate cytotoxicity on pediatric cancer D283 medulloblastoma cells.

**Figure 1:**
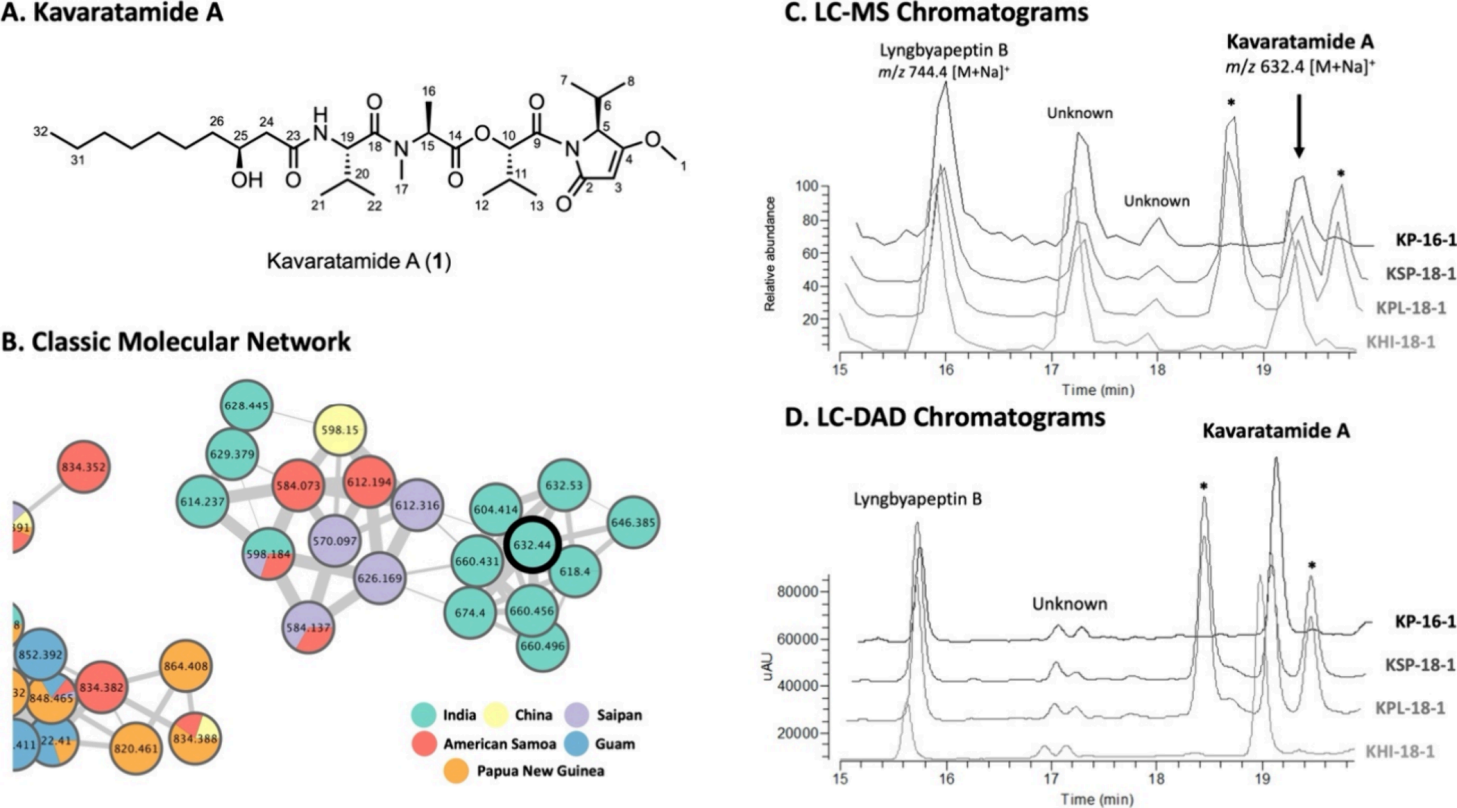
(A) Chemical structure of kavaratamide A (**1**). (B) Kavaratamide cluster in the molecular network of *M*. *bouillonii* extracts from six different regions, including India (green), China (yellow), Saipan (purple), American Samoa (red), Guam (blue), and Papua New Guinea (orange). Kavaratamide A is the black bold node from India at *m*/*z* 632. (C) Enlarged MS base peak chromatogram (retention time 15.0–19.8 min) of *M*. *bouillonii* collected from India. (D) Enlarged DAD chromatogram (retention time 15.0–19.8 min, 200–600 nm) of *M*. *bouillonii* collected from India. *Peaks identified as chlorophyll derivatives based on their UV/vis spectra.

## Results and Discussion

Previously, we used the ORCA tool and classical molecular networking to discover and characterize region-specific metabolites from a marine cyanobacterium.^20^ Fifteen extracts of *M*. *bouillonii* were sourced from six distinct regions, India (Kavaratti, Lakshadweep Islands), China (Xisha, Paracel Islands), Saipan, American Samoa, Guam, and Papua New Guinea, and led to the isolation and structural characterization of the doscadenamides as metabolites specific to Saipan among the data sets. In the current study, we focused on *M*. *bouillonii* specimens collected from the Lakshadweep Islands of India, an underexplored area in previous marine natural products research.

First, the LC-MS/MS files of the 15 *M*. *bouillonii* samples used in the previous study were used to construct a classical molecular network in the GNPS ecosystem and visualized with Cytoscape (Figure 1B).^20^ To improve the efficiency of targeting, isolating, and characterizing compounds distinctive of a particular region, each node within the network was assigned a different color, corresponding to the geographical location of its collection (Figure 1B).

However, because of the high sensitivity of MS analyses and the correspondingly high detectability of compounds by GNPS analyses, it can be difficult in practice to isolate minor compounds observed only by these techniques. Incorporating manual inspection of LC-MS and LC-DAD chromatograms represents a pragmatic solution to this dilemma. DAD chromatograms, in particular, can offer a more reliable assessment of the relative abundance of metabolites in the analyzed sample, especially if there is a conserved chromophore in the structures of the analogues. Through investigation of the LC-MS and LC-DAD chromatograms of four extracts of *M*. *bouillonii* collected from the Lakshadweep Islands in 2016 and 2018, we identified two peaks that appeared consistently across all four samples. These were accompanied by several chlorophyll-type pigments, recognized as such by their UV absorption close to 400 nm and retention time (Figure 1C and D). One of the two interesting peaks exhibited a sodium adduct ion at *m*/*z* 744.4 [M + Na]^+^ (*t*_R_ 15.9 min) (Figures 1B–D, S2) and was subsequently identified as lyngbyapeptin B through a dereplication search using the MarinLit database (https://marinlit.rsc.org/). The second interesting peak, which was unique to the India collections by ORCA analysis, showed a sodium adduct ion at *m*/*z* 632.4 [M + Na]^+^ (*t*_R_ 19.1 min) (Figure 1B–D), and dereplication efforts were unsuccessful in identifying this as a known compound. Therefore, it was prioritized for isolation and the structure determination. The alcohol-preserved biomass of the three 2018 cyanobacterial collections was extracted with 2:1 CH_2_Cl_2_/MeOH, and following a CH_2_Cl_2_/H_2_O partition and thorough drying, the lipophilic extracts of all three showed very similar LC-MS chemical profiles. The three extracts were combined and separated into nine fractions (A–I) using normal-phase medium-pressure liquid chromatography. LC-MS analysis of all nine fractions revealed that fraction G contained the target metabolite. This fraction was subjected to RP-HPLC to afford this compound, given the common name kavaratamide A (**1**) (Figure 1A).

**Figure 2:**
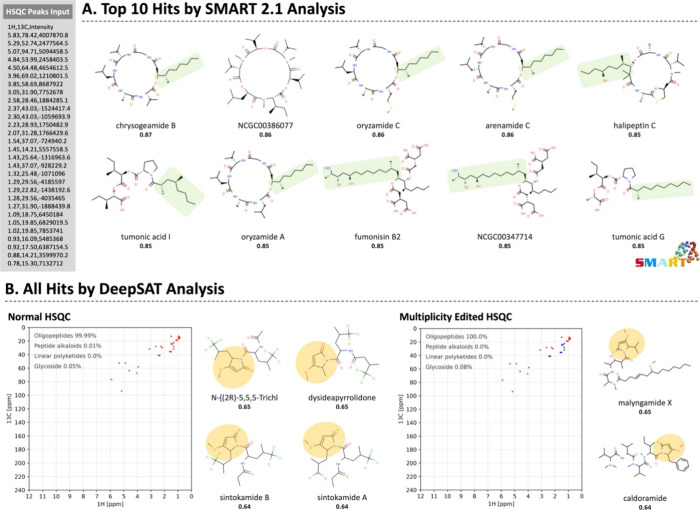
Results of ^**1**^H–^13^C HSQC-based analysis of kavaratamide A (**1**) using (A) SMART 2.1 and (B) DeepSAT. The cosine score is shown under the name of each compound. The highlighted sections roughly match portions of kavaratamide A.

Kavaratamide A (**1**), a white amorphous solid, had a molecular formula of C_32_H_55_N_3_O_8_ by HRESIMS. Small molecule accurate recognition technology (SMART), an artificial intelligence (AI)-based tool, was used to generate structural hypotheses from ^1^H–^13^C HSQC data of **1**.^22^ The list of correlation peaks obtained from the multiplicity edited ^1^H–^13^C HSQC spectrum of **1** was utilized in SMART 2.1 (http://smart.ucsd.edu) to provide the top 10 hits. These were a mixture of cyclic lipodepsipeptides, cyclic depsipeptides, and linear lipodepsipeptides (Figure 2A). SMART 2.1 employs a deep convolutional neural network (CNN) that was trained with a combination of real data and calculated ^1^H–^13^C HSQC spectra (ACD Laboratories) of established compounds. The continual expansion of the ^1^H–^13^C HSQC spectrum library in SMART 2.1 is paramount to improving its structure predictions. However, the limited availability of ^1^H–^13^C HSQC spectra for numerous compounds and the protracted effort required for accurate calculation of ^1^H–^13^C HSQC spectra have posed hindrances to the expansion of SMART 2.1.

Recently, we introduced DeepSAT (https://deepsat.ucsd.edu), an enhanced structure annotation tool utilizing ^1^H–^13^C HSQC NMR spectra in a different way.^23^ DeepSAT leverages a CNN-based multitask supervised learning architecture that was trained to predict Morgan fingerprints from the input ^1^H–^13^C HSQC spectra. As Morgan fingerprints are easily created for all established compounds, there is a great expansion of the number of compounds available for comparison, despite there being no significant difference in the number of ^1^H–^13^C HSQC spectra utilized for the training of these two tools. As these two annotation tools were trained in different ways to predict related structures, we anticipated that running both tools could be insightful and possibly provide complementary results.

Normal and multiplicity edited ^1^H–^13^C HSQC data obtained for compound **1** were applied to DeepSAT analysis, and there were clear differences from those obtained by SMART 2.1. Both normal and multiplicity edited ^1^H–^13^C HSQC analyses identified the closest matches with oligopeptides containing an *O*-methyl 2-substituted pyrrolinone unit (Figure 2B). With clues obtained by these SMART 2.1 and DeepSAT analyses, we initiated a detailed structure elucidation of **1** with the hypothesis that it was a lipodepsipeptide with an *O*-methyl 2-substituted pyrrolinone moiety.

The ^1^H NMR data of **1** (Table 1) had two deshielded singlet methyl signals at δ_H_ 3.85 and 3.04 indicative of *O*- and *N*-methyl moieties, respectively. A triplet methyl proton signal (δ_H_ 0.88) and seven aliphatic proton signals (δ_H_ 2.38–1.24) indicated the presence of an aliphatic chain. Seven doublet methyl signals (δ_H_ 1.46, 1.09, 1.05, 1.01, 0.92, 0.91, and 0.78) along with three septet methine protons signals (δ_H_ 2.56, 2.23, and 2.06) and one quartet α-amino methine proton signal (δ_H_ 5.29) indicated the presence of three isopropyl moieties and one secondary methyl group. These were accompanied by two doublet α-amino methine proton signals (δ_H_ 4.84 and 4.50) and a carbinol proton signal (δ_H_ 5.82, δ_C_ 78.4, and C-10) that indicated the presence of amino acids or organic acids with corresponding amide or ester moieties. This was reinforced by the presence of five amide or ester carbonyl carbons (δ_C_ 170.2, 169.1, 171.1, 172.3, and 172.9) in the ^13^C NMR spectrum. Also present were signals for an olefinic proton (δ_H_ 5.07), a second carbinol methine proton (δ_H_ 3.95, δ_C_ 69.0, C-25), an amide proton (δ_H_ 6.54), and two distinctive olefin carbons (δ_C_ 180.0 and 94.7) along with a number of shielded aliphatic carbon resonances (see Table 1).

**Table 1:** ^1^H (500 MHz) and ^13^C NMR (125 MHz) Spectroscopic Data for Kavaratamide A (**1**) in CDCl_3_^a^

residue	position	δ_C_, type	δ_H_ (*J*in Hz)	COSY	HMBC
**iPr-***O***-Me-Pyr**	**1**	58.7, CH_3_	3.85, s	-	4
	**2**	170.2, C	-	-	-
	**3**	94.7, CH	5.07, s	-	2, 5
	**4**	180.0, C	-	-	-
	**5**	64.4, CH	4.50, d (2.5)	6	2, 4, 6, 7, 8
	**6**	28.5, CH	2.56, sepd (7.5, 2.5)	5, 7, 8	4, 5, 7, 8, 9^b^
	**7**	18.8, CH_3_	1.09, d (7.5)	6	5, 6, 8
	**8**	15.3, CH_3_	0.78, d (7.0)	6	5, 6, 7
**Hiva**	**9**	169.1, C	-	-	-
	**10**	78.4, CH	5.82, d (3.5)	11	9, 11, 12, 13, 14^b^
	**11**	28.9, CH	2.23, sepd (6.5, 3.0)	10, 12, 13	12
	**12**	19.8, CH_3_	1.05, d (6.5)	11	10, 11, 13
	**13**	16.1, CH_3_	0.92, d (7.0)	11	10, 11, 12
*N***-Me-Ala**	**14**	171.1, C	-	-	-
	**15**	52.8, CH	5.29, q (7.0)	16	14, 16, 17, 18^b^
	**16**	14.2, CH_3_	1.46, d (7.0)	15	14, 15
	**17**	31.9, CH_3_	3.04, s	-	15, 18
**Val**	**18**	172.3, C	-	-	-
	**19**	53.9, CH	4.84, dd (8.5, 5.5)	20, 19-NH	18, 20, 21, 22, 23
	**20**	31.3, CH	2.06, sep (7.0)	19, 21, 22	19, 21, 22, 23^b^
	**21**	19.8, CH_3_	1.01, d (7.0)	20	19, 20, 22
	**22**	17.4, CH_3_	0.91, d (6.5)	20	19, 20, 21
	**19-NH**	-	6.54, d (8.5)	19	23
3-HDA	**23**	172.9, C	-	-	-
	**24a**	42.9, CH_2_	2.38, dd (15.0, 2.5)	24b, 25	23, 25, 15
	**24b**		2.29, dd (15.0, 9.5)	24a, 25	23, 25, 15
	**25**	69.0, CH	3.95, br s	24a, 24b, 26a, 26b	-
	**26a**	37.1, CH_2_	1.54, dd (17.0, 7.5)	-d	-d
	**26b**		1.42, overlap	-d	-d
	**27**	25.6, CH_2_	1.43–1.24, m	-d	-d
	**28**	29.7^c^, CH_2_	1.43–1.24, m	-d	-d
	**29**	29.4^c^, CH_2_	1.43–1.24, m	-d	-d
	**30**	31.9, CH_2_	1.43–1.24, m	-d	-d
	**31**	22.8, CH_2_	1.43–1.24, m	-d	-d
	**32**	14.3, CH_3_	0.88, t (7.0)	31	30, 31

a HSQC data acquired at 600 MHz.

b Weak signals.

c Chemical shifts are interchangeable.

d The correlations were observed but not positionally identified due to overlapped signals (δ_H_ 1.43–1.24 ppm).

**Figure 3:**
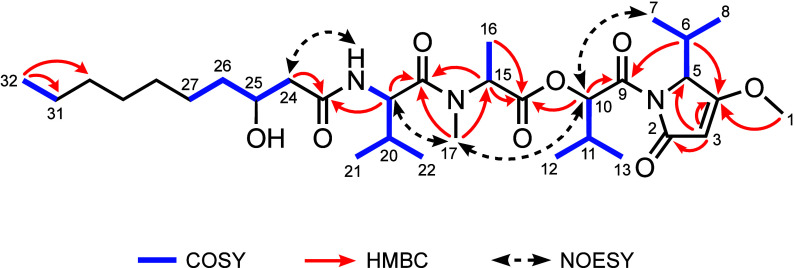
Key COSY, HMBC, and NOESY correlations of kavaratamide A (**1**).

**Figure 4:**
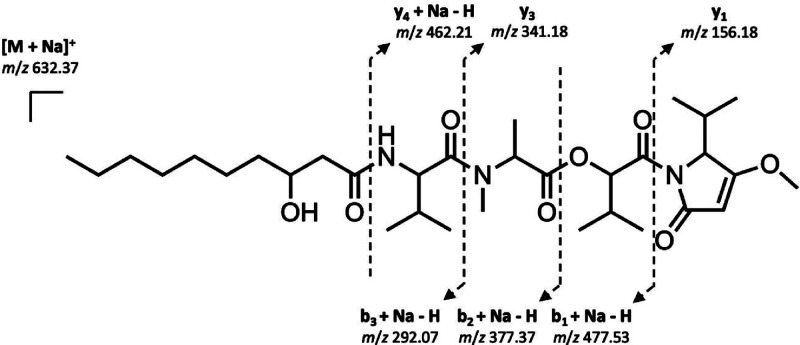
Key MS/MS fragments observed for kavaratamide A (**1**).

A detailed analysis of the COSY and HMBC data allowed construction of the complete planar structure of kavaratamide A (**1**) (Figure 3). COSY correlations between H-10/H-11/H_3_-12/H_3_-13, H-15/H_3_-16, and 19-NH/H-19/H-20/H_3_-21/H_3_-22, along with HMBC correlations of H-10 with C-9/C-14, H-15 with C-14/C-18, H_3_-17 with C-15/C-18, and H-19 with C-18, established the hydroxyisovaleric acid (Hiva), *N*-Me-alanine (*N*-Me-Ala), and valine (Val) moieties, and these were sequentially connected through ester and amide bonds. The COSY correlations between H-5/H-6/H_3_-7/H_3_-8 along with HMBC correlations between H_3_-1/C-4 and H-3/C-2/C-4/C-5 allowed the formulation of the isopropyl-*O*-Me-pyrrolinone (iPr-*O*-Me-pyr) system. This moiety was connected to the Hiva residue through HMBC correlations between H-6/C-9 and H-10/C-9. COSY correlations between H_2_-24/H-25/H_2_-26/H_2_-27 and H_2_-31/H_3_-32, HMBC correlations between H_3_-32 and C-31/C-30, and the remaining unassigned two methylene aliphatic carbons (δ_C_ 29.7 and 29.4; C-28 and C-29) suggested the presence of a terminating 3-hydroxydecanoic acid residue (3-HDA). HMBC correlations between H-19/C-23 and H_2_-24/C-23 positioned the valine residue adjacent to the 3-HDA group. This linkage was also supported by NOESY correlations of H_3_-7 with H-10, H-10 with H_3_-17, H_3_-17 with H-19, and 19-NH with H_2_-24 (Figure 3). Taken together, the planar structure was deduced as a linear lipodepsipeptide consisting of iPr-*O*-Me-pyr–Hiva–*N*-Me-Ala–Val–3-HDA. MS fragment ions detected in the MS and MS/MS spectra of pure kavaratamide A (**1**) (*m*/*z* 477.53, 462.21, 377.37, 341.18, 292.07, and 156.18) provided additional support for the overall linear structure and sequence of linked moieties (Figure 4).

**Figure 5:**
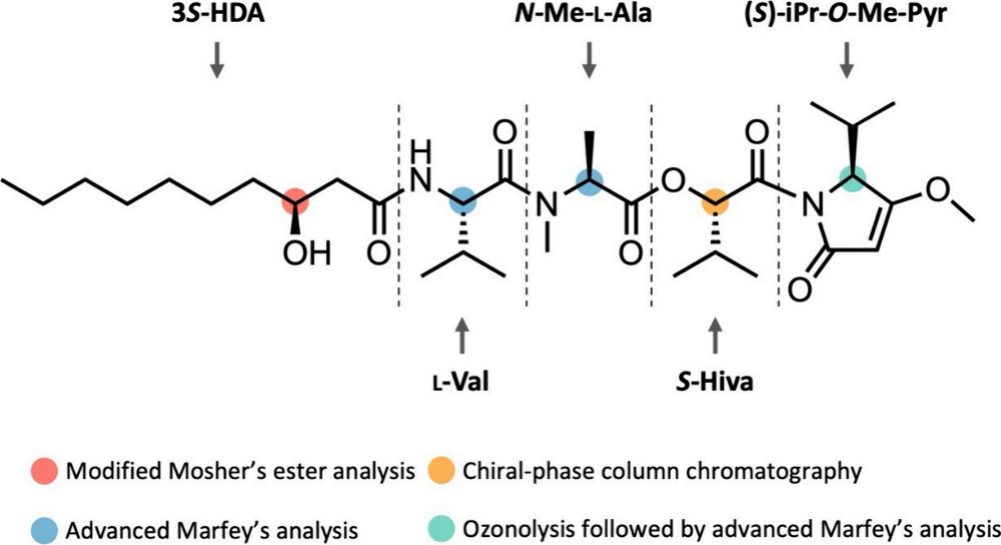
Absolute configuration analysis of kavaratamide A (**1**).

To determine the absolute configuration of kavaratamide A (**1**), several analytical techniques were employed, including ozonolysis, acid hydrolysis followed by advanced Marfey’s analysis, modified Mosher’s ester analysis, and chiral-phase HPLC (Figure 5). Ozonolysis, followed by acid hydrolysis, yielded 3-HDA, Val-1, *N*-Me-Ala, Hiva, and Val-2 (derived from iPr-*O*-Me-pyr). 3-HDA, the sole lipophilic product among the five partial structures produced by acid hydrolysis, was extracted after acid hydrolysis by solvent–solvent partitioning between CH_2_Cl_2_ and water. We used Mosher’s ester analysis for determining the absolute configuration at C-3 of the 3-HDA residue which was recovered from the lipophilic phase. Preliminary Mosher’s esterification experiments with authentic 3-HDA provided only a low yield of the MTPA ester, suggesting that it would be challenging to apply the typical NMR chemical shift analysis of the (*R*)-MTPA ester and (*S*)-MTPA ester. Therefore, we analyzed the Mosher’s esters using an HPLC-UV-MS analysis in comparison with authentic standards and determined the absolute configuration at C-25 in 3-HDA to be *S* (Figure S10). Next, a portion of the water layer was injected onto a chiral-phase column, and the retention time of Hiva derived from kavaratamide A (**1**) was compared with those of authentic *S*- and *R*-Hiva. For Val-1, Val-2, and *N*-Me-Ala, the remaining water layer was derivatized with Marfey’s reagent, and the retention times were compared with authentic l- and d-forms of these two amino acids. These analyses identified the absolute configurations at C-10 in Hiva, at C-15 in *N*-Me-Ala, and at C-19 in the Val residue to be *S*, l-, and l-, respectively. Additionally, the absolute configuration at C-5 in the iPr-*O*-Me-pyr residue was also determined to be *S*, as only the l-form of valine was identified by Marfey’s analysis (Figure S11).

**Figure 6:**
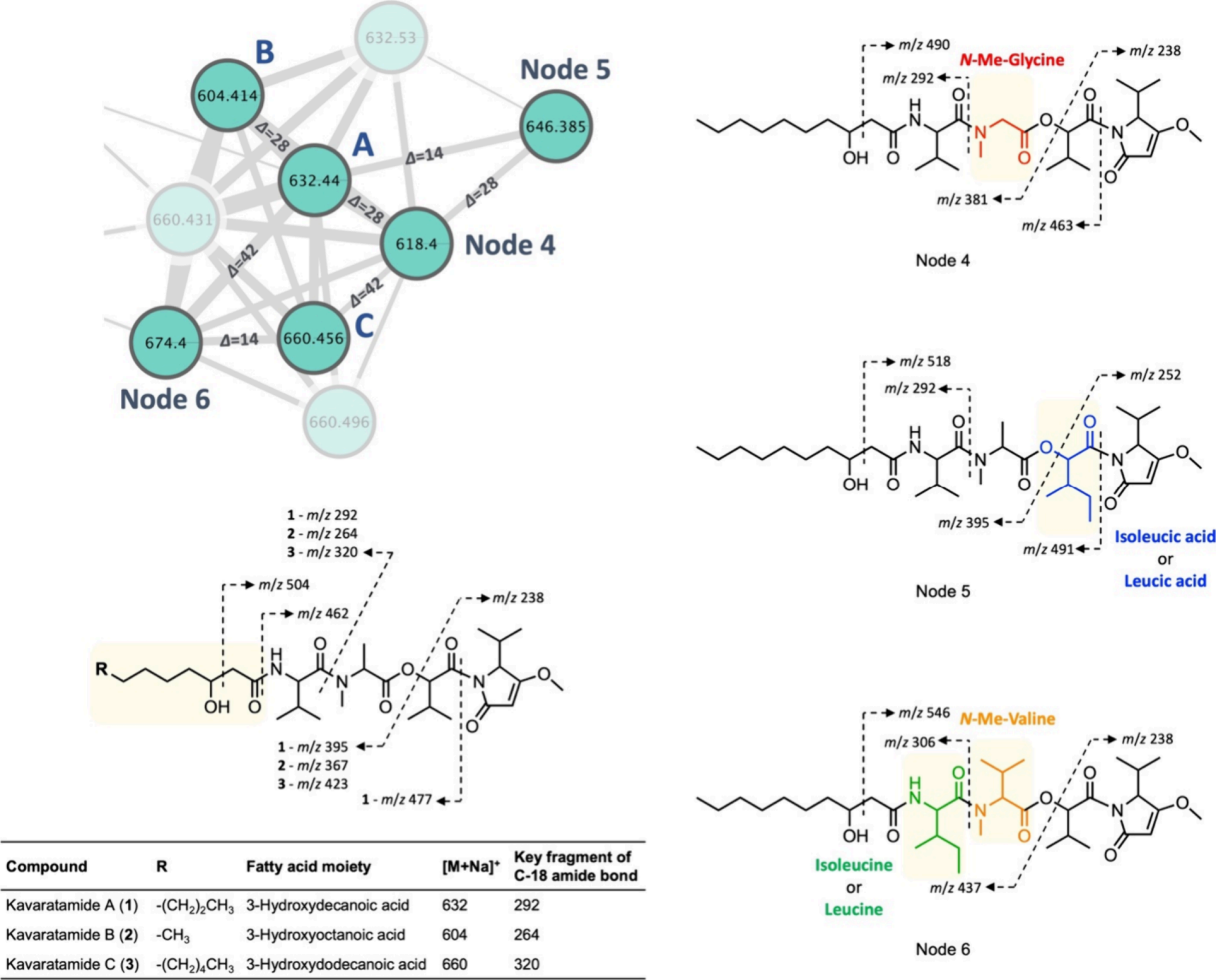
Proposed structures of the analogues of kavaratamide A (**1**) and their key MS/MS fragmentation. In the molecular network, the blurred nodes were identified as identical compounds with either *m*/*z* 632 (kavaratamide A) or *m*/*z* 660 (kavaratamide C) by a comparison of retention time and MS/MS fragment alignment. Solid nodes are labeled ‘A’ for kavaratamide A (**1**), ‘B’ for kavaratamide B (**2**), and ‘C’ for kavaratamide C (**3**). The light-yellow-highlighted sections on the structures highlight the structural differences from those of kavaratamide A.

The elucidation of the complete structure of kavaratamide A (**1**) facilitated an examination of its MS/MS fragmentation patterns deriving from the sodium adduct ion at *m*/*z* 632. As expected, most of the fragments arose from cleavage of the amide or ester bonds (e.g., *m*/*z* 477, 395, 292, 263, and 238); however, there were also a few other insightful cleavages, such as a McLafferty rearrangement cleaving C-24–C-25 to generate *m*/*z* 504 (Figures 6 and S18). As a result of this analysis, several analogues could be identified by MS-fragmentation data from the same cyanobacterial collection that yielded kavaratamide A (**1**). These had parent masses of *m*/*z* 674, 660, 646, 632 (=compound **1**), 618, and 604, with the mass differences between each of them of 14 Da, indicative of the gain or loss of CH_2_ in each case. For the parent ions at *m*/*z* 604 and 660, the presence of fragments at *m*/*z* 504 and 462 in the MS/MS spectrum suggested structural variations within the 3-hydroxy fatty acid moiety, namely, the gain or loss of two methylene groups relative to kavaratamide A (**1**). Thus, kavaratamides B (**2**, *m*/*z* 604) and C (**3**, *m*/*z* 660) are proposed as kavaratamide A analogues possessing the acyl groups 3-hydroxyoctanoic acid and 3-hydroxydodecanoic acid, respectively. This deduction is conceptually supported by several reports on cyanobacterial lipopeptides and their analogues that are only different in the length of their fatty acid chains.^24,25^ For nodes 4–6 (*m*/*z* 618, 646, and 674), the presence of fragments at *m*/*z* 490, 518, and 546, respectively, rather than *m*/*z* 504 in each suggested that structural modifications were present within the amino acid sequence and not the fatty acid acyl group. The observation of fragment ions *m*/*z* 292 and 381 from the parent ion *m*/*z* 618, corresponding to node 4, indicated the loss of a methyl group from the *N*-Me-Ala residue, suggesting it was replaced by either Ala or *N*-Me-glycine (*N*-Me-Gly). Based on the biosynthetic pathway inferred from the complete structure of kavaratamide A (**1**), the module responsible for incorporation of *N*-Me-Ala contains an active *N*-methylation domain. Therefore, it is probable that the substituted amino acid in compound 4 also possesses an *N*-methyl group. Moreover, Gly commonly replaces Ala in NRPS peptides as they are both small, nonpolar amino acids. Consequently, the substituted amino acid sequence in compound no. 4 is likely *N*-Me-Gly. The structure of node 5 (*m*/*z* 646) is proposed to be similar to kavaratamide A with the key distinction being the replacement of the Hiva residue with an isoleucic acid (or leucic acid), supported by the observation of MS fragment ions *m*/*z* 491, 395, and 252. Node 6 (*m*/*z* 674) had MS fragment ions at *m*/*z* 437, 306, and 238, suggesting two structural differences: replacement of the Val residue by Ile (or Leu) and replacement of the *N*-Me-Ala residue by *N*-Me-Val. From LC-MS data, the sums of the intensities of the precursor ions of each compound were used to deduce their relative abundances as follows: **1** > node 4 > **3** > node 5 > **2** > node 6 (Figure S20). Although kavaratamide A analogues **2** and **3** and nodes 4–6 were detected in the LCMS chromatogram, their isolation was unsuccessful because they were present in very low quantities in the extract. We propose structures of the analogues through detailed MS/MS fragment interpretation of each; however, further isolation work is necessary to define their complete structures.

iPr-*O*-Me-pyr is a relatively rare structural unit in natural products. The only known cases of natural products possessing this moiety include the mirabimides from the cyanobacterium *Scytonema mirabile*,^26^ malyngamide X from the sea hare *Bursatella leachii*,^27^ and iheyamide A from the cyanobacterium *Dapis* sp.^28^ Observation of antitrypanosomal activity of the iheyamides A–C against *Trypanosoma brucei rhodesiense* and *T*. *b*. *brucei* revealed that iPr-*O*-Me-pyr may be a potential pharmacophore.^28^ A subsequent synthetic study on antitrypanosomal activity associated with the iPr-*O*-Me-pyr moiety confirmed the importance of not only the iPr-*O*-Me-pyr moiety but also the length of the peptide chain.^29^ As a result, we screened kavaratamide A (**1**) for activity against *T*. *b*. *brucei* at concentrations ranging from 4 to 10 μM. We also screened it against the parasite proteolytic enzymes, *T. brucei* cathepsin L-like protease (*Tbr*CATL),^30^ and the related protease, cruzain (CRZ), from *T. cruzi*,^31^ both of which are validated targets for small-molecule therapeutics.^32^ However, kavaratamide A (**1**) did not inhibit the growth of the parasite or the activity of the proteases (Table S2). Iheyamide A comprises a peptide structure featuring an iPr-*O*-Me-pyr moiety followed by nonpolar amino acids, and a synthetic study established that greater activity correlated with an increased length of the peptide chain of nonpolar amino acids attached to the iPr-*O*-Me-pyr moiety.^28,29^ The absence of activity observed with kavaratamide A (**1**) in our assays may be attributed to the 3-hydroxy group in the 3-HDA residue that imparts a polar characteristic to this section of the compound. We also evaluated kavaratamide A (**1**) for cytotoxicity to the human medulloblastoma D283-med cell line, which revealed moderate cytotoxicity with a CC_50_ value of 7.1 ± 0.3 μM.

In summary, we report here on a new linear lipodepsipeptide, kavaratamide A (**1**), and two of its analogues, kavaratamides B and C (**2**, **3**), from the cyanobacterium *M*. *bouillonii* collected near Kavaratti, India. Kavaratamide A (**1**) was prioritized for isolation and characterization using a comparative chemogeographic analysis, including usage of the ORCA tool.^20^ The type of structure was suggested using the AI-based tools SMART 2.1 and DeepSAT, and its planar structure was rigorously determined by comprehensive NMR analysis and HRMS and LR-MS/MS-fragmentation interpretation. A variety of methods for determination of the absolute configuration were used, including chemical degradation, advanced Marfey’s analysis, modified Mosher’s ester analysis, and chiral-phase column chromatography, which led to the complete absolute configuration of kavaratamide A (**1**). This is the first study to explore the natural products of the cyanobacterium *M*. *bouillonii* from India. These results support the hypothesis that a chemogeographical approach for discovering new bioactive natural products can be productive.

## Experimental Section

### General Experimental Procedures

Optical rotation was measured on a Jasco P-2000 polarimeter using a 1 cm microcell (JASCO International Co. Ltd.). UV and IR spectra were recorded on Beckman Coulter DU-800 (Beckman Coulter Life Sciences) and Nicolet iS50 FT-IR spectrometers (Thermo Fisher Scientific, Inc.), respectively. 1D and 2D NMR spectra were acquired at room temperature using a Bruker Avance III DRX-600 NMR with a 1.7 mm dual tune TCI cryoprobe and a JEOL ECZ 500 MHz NMR spectrometer equipped with a 3 mm inverse detection probe. NMR spectra were referenced to residual solvent CDCl_3_ signals (δ_H_ 7.26 and δ_C_ 77.16 as internal standards). NMR spectra were analyzed using MestReNova v 14.3.0-30573 (Mestrelab). LR-LCMS data were collected on a Thermo Finnigan Surveyor Autosampler/LC-Pump-Plus/PDA-Plus with a Thermo Finnigan Advantage Max mass spectrometer equipped with a Kinetex 5 μ C18 100 Å analytical column (100 × 4.6 mm, 5 μm, Phenomenex). All low-resolution LC-MS/MS data were analyzed using Xcalibur Qual Browser v. 1.4 SR1 (Thermo Fisher Scientific, Inc.). Medium-pressure liquid chromatography (MPLC) was performed on a Teledyne ISCO CombiFlash Rf using RediSep Gold 80 g HP silica columns. Column chromatography was performed using C18 solid-phase extraction (SPE) with 1 g of Bond Elut-C18. Analytical and semipreparative HPLC purification was carried out with a Thermo Scientific Dionex UltiMate 3000 LC system interfaced to a DAD detector (Dionex, Thermo Fisher Scientific Company) equipped with a Kinetex 5 μ C18 100 Å analytical column (100 × 4.6 mm, 5 μm, Phenomenex) or a Kinetex 5 μ C18 semipreparative column (150 × 100 mm, 5 μm, Phenomenex) using Chromeleon software. An Agilent 6230 time-of-flight mass spectrometer (TOFMS) with a Jet Stream electrospray ionization source (ESI) was used for high-resolution mass spectrometry analysis. The Jet Stream ESI source was operated under positive ion mode with the following parameters: VCap: 3500 V; fragmenter voltage: 160 V; nozzle voltage: 500 V; drying gas temperature: 325 °C; sheath gas temperature: 325 °C; drying gas flow rate: 7.0 L/min; sheath gas flow rate: 10 L/min; nebulizer pressure: 40 psi. Solvents used for extraction, purification, and LC-MS/MS analysis were purchased from Fisher Chemical. All solvents were HPLC or LC-MS grade. Deuterated solvents were purchased from Cambridge Isotope Laboratories.

### Cyanobacterial Collection and Taxonomy

The benthic filamentous tropical marine cyanobacterium *Moorena bouillonii* was collected via snorkeling in Kavaratti, Lakshadweep Islands, India; KP-16-1 collected on February 6, 2016; KSP-18-1, KPL-18-1, and KHI-18-1 collected on April 7–8, 2018). Samples from all locations were preserved in 1:1 seawater and either EtOH or isopropyl alcohol and stored frozen until extraction in the laboratory. The organisms were identified as red-filamentous *M*. *bouillonii* that were woven into sheets by the weaver shrimp *Alpheus frontalis* on the basis of morphological characteristics.^33^ For details about collection records of these samples, see Supporting Information (Table S1).

### GNPS Classical Molecular Networking

All LC-MS/MS .raw files were converted to .mzXML by using a complete package for Windows OS, following the description on the GNPS Web site (https://ccms-ucsd.github.io/GNPSDocumentation/fileconversion/). A molecular network was created using the online workflow (https://ccms-ucsd.github.io/GNPSDocumentation/) on the GNPS Web site (http://gnps.ucsd.edu) using the converted .mzXML files.^21^ The data were filtered by removing all MS/MS fragment ions within ±17 Da of the precursor *m*/*z*. MS/MS spectra were window filtered by choosing only the top 6 fragment ions in the ±50 Da window throughout the spectrum. The precursor ion mass tolerance was set to 2.0 Da, and an MS/MS fragment ion tolerance was set to 0.5 Da. A network was then created where edges were filtered to have a cosine score above 0.7 and more than 4 matched peaks. Further, edges between two nodes were kept in the network only if each of the nodes appeared in each other’s respective top 10 most similar nodes. Finally, the maximum size of a molecular family was set to 100, and the lowest scoring edges were removed from molecular families until the molecular family size was below this threshold. The spectra in the network were then searched against GNPS spectral libraries. The library spectra were filtered in the same manner as the input data. All matches kept between network spectra and library spectra were required to have a score above 0.7 and at least 4 matched peaks. The generated molecular networks were visualized using Cytoscape (version 3.9.1).^34^

### Extraction and Isolation

The biomass of each cyanobacterial *M*. *bouillonii* collection was thoroughly extracted with 2:1 CH_2_Cl_2_ and MeOH. After concentration of the extracts under vacuum, LC-MS/MS samples were prepared by resuspension in MeOH at a concentration of 1 mg/mL followed by elution through C18 SPE cartridges. The three collections from 2018 were combined because their LC-MS profiles were essentially identical except for pigment peaks (identified by the presence of UV absorption maxima above 400 nm) (Figures S3 and S4). The combined extracts (KSP-18-1, 4.6 g; KPL-18-1, 2.0 g; KHI-18-1, 5.71 g; extract weights included carry-through salts) were fractionated into nine fractions (A–I) by normal-phase medium-pressure liquid chromatography, eluted with a stepwise gradient [*n*-hexanes/EtOAc/MeOH = 100/0/0 (fr. A), 90/10/0 (fr. B), 80/20/0 (fr. C), 60/40/0 (fr. D), 40/60/0 (fr. E), 20/80/0 (fr. F), 0/100/0 (fr. G), 0/75/25 (fr. H), and 0/0/100 (fr. I); 300 mL each]. Fraction G (7.2 mg) was subjected to HPLC (Kinetex 5 μm C18, 150 × 10.0 mm, 70–80% MeCN in H_2_O containing 0.1% formic acid, flow rate: 3.0 mL/min) and afforded kavaratamide A (**1**, 1.5 mg, *t*_R_ 26.2 min).

#### Kavaratamide A (**1**)

White amorphous powder; [α]_D_^25^ −17 (*c* 1.0, MeOH); UV (MeOH) λ_max_ (log ε) 238 (3.53) nm; IR (ATR) ν_max_ 3317, 2959, 2921, 2851, 1728, 1695, 1622, 1464, 1388, 1342, 1318, 1249, 1204, 1137, 1091, 995, 939, 808, 746, 646 cm^–1^; ^1^H and ^13^C NMR data, Table 1; HRESIMS *m*/*z* 610.4071 [M + H]^+^ (calcd for C_32_H_56_N_3_O_8_, 610.4062).

### Ozonolysis and Acid Hydrolysis

Kavaratamide A (**1**, 0.2 mg) was dissolved in 1 mL of CH_2_Cl_2_ and ozonized at −78 °C for 10 min, and the resulting ozonide detected by LR-LCMS at *m*/*z* 658.38. The solvent was evaporated under a stream of N_2_, and the product was treated with 6 N HCl (1 mL) in a sealed vial for 16 h at 80 °C. The dried hydrolysate (0.2 mg) was suspended in H_2_O (1 mL) and partitioned with CH_2_Cl_2_ (1 mL) four times. The CH_2_Cl_2_ layer was dried under a N_2_ stream for further analysis, and the water layer was directly subjected to chiral-phase HPLC analysis (see below).

### Modified Mosher’s Ester Analysis for 3-HDA

The absolute configuration of 3-hydroxydecanoic acid was determined using a modified Mosher’s ester analysis.^35^ After transferring the CH_2_Cl_2_ layer into a 1.5 mL vial followed by evaporation under a stream of N_2_, anhydrous pyridine (1 μL), *R*-MTPA-Cl (1 μL), and CDCl_3_ (200 μL) were sequentially added to the vial. The mixture was stirred for 2 h at room temperature (rt). Authentic samples of 3*R*-HDA (0.5 mg, Avanti Polar Lipids) and (*rac*)-3-HDA (0.8 mg, Sigma-Aldrich) were derivatized in the same way. All samples were dried under N_2_ followed by dissolution in MeOH and analysis by LR-LCMS equipped with a PDA-Plus detector (Thermo Fisher Scientific). The samples were injected onto a Kinetex 5 μ C18 100 Å analytical column (100 × 4.6 mm, 5 μm, Phenomenex) using a mixture of MeCN and H_2_O containing 0.1% formic acid (gradient methods; 50/50 to 70/30 MeCN/H_2_O for 17 min; flow rate 0.75 mL/min). The retention times for the (*S*)-MTPA-3*R*-HDA ester and (*S*)-MTPA-3*S*-HDA ester were 14.00 and 14.32 min, respectively. The retention time of the (*S*)-MTPA-3-HDA ester from kavaratamide A (**1**) was 14.32 min, indicating that it was 3*S*-HDA.

### Chiral-Phase Column Chromatography and Advanced Marfey’s Analysis for Organic Acids

The water layer was analyzed by chiral-phase column chromatography with UV detection at 254 nm in comparison with the retention times of authentic *R*- and *S*-Hiva (purchased from Bachem and Chem-Impex International, respectively) [Phenomenex Chirex 3126 (D), 4.6 × 250 mm; mixture of ACN and 2 mM CuSO_4_ in H_2_O (15/85) at 1.0 mL/min; detection at 254 nm]. The retention times for *R*- and *S*-Hiva were 10.49 and 7.71 min, respectively. The retention time of Hiva from kavaratamide A (**1**) was 7.71 min, indicating *S*-Hiva.

After the chiral-phase column analysis, the remaining sample from the water layer was subject to Marfey’s analysis. The sample was dried and resuspended in H_2_O (100 μL) followed by the addition of a 1% *N*-α-(2,4-dinitro-5-fluorophenyl)-l-valinamide (l-FDVA) acetone solution (500 μL), 1 M NaHCO_3_ (100 μL), and DMSO (50 μL). Authentic samples including d-Val, dl-Val, *N*-Me-l-Ala, and *N*-Me-dl-Ala were prepared as 50 mM solutions (each 50 μL in 5 mL vials), and a 1% l-FDVA acetone solution (100 μL), 1 M NaHCO_3_ (20 μL), and DMSO (10 μL) were added. The mixtures were stirred at 40 °C for 1 h and then cooled to rt, and 2 M HCl (50 μL for the kavaratamide A-derived sample, 10 μL for the authentic samples) was added to each reaction. All samples were dried under a stream of N_2_, and 1 mL MeOH was added to the kavaratamide A sample and 10 mL of MeOH added to the authentic samples. After filtration through a nylon filter (0.2 μm), all of the samples were subjected to analytical HPLC analysis (Kinetex 5 μ C18 100 Å analytical column, 100 × 4.6 mm, 5 μm) using a linear gradient (30/70 to 45/55 MeCN/H_2_O containing 0.1% formic acid for 20 min, 0.8 mL/min, UV detection at 340 nm). The retention times for the derivatized standard amino acids were as follows: d-Val (17.50 min), l-Val (11.26 min), *N*-Me-d-Ala (9.51 min), and *N*-Me-l-Ala (9.21 min). Only l-Val and *N*-Me-l-Ala were observed in the kavaratamide A-derived (**1**) sample, thus indicating that the Val residue as well as the Val residue released from the iPr-*O*-Me-pyr moiety, and the *N*-Me-Ala residue, were all of the l configuration. Therefore, C-5 in the iPr-*O*-Me-pyr moiety is of the *S* configuration.

### Cytotoxicity Assays on D283-med Cells for Kavaratamide A

A 48 h viability assay with D283-med (medulloblastoma) cells (ATCC) was used to evaluate the cytotoxic activity of kavaratamide A. Suspension cells in complete EMEM medium were seeded at 1.8 × 10^5^ cells/mL into white 96-well plates and after 1 h exposed to 10 dilutions of kavaratamide A starting from 50 μM. The total assay volume was 100 μL/well, and quisinostat served as the positive control (CC_50_ = 11 ± 2 nM). DMSO was used as the initial dissolving solvent for the test compounds, and its concentration in the wells was kept at less than 0.5% (v/v). Cells were lysed with 90 μL/well of reconstituted CellTiter-Glo reagent (Promega G7572), and the resulting luminescence was measured on a SpectraMax M3 microplate reader (Molecular Devices). Concentration–response curves were created with log(inhibitor) vs response with the variable slope (four parameters) logistic model, and their CC_50_ values were calculated using GraphPad Prism version 10.1.2 for Windows (Figure S21).

## Data Availability

The NMR data for compound **1** have been deposited in the Natural Products Magnetic Resonance Database (np-mrd.org) under accession number NP0332591.^36^

## Supporting Information Available

Detailed sample collection records, LC-MS and LC-DAD chromatograms of four *M*. *bouillonii* samples collected from Kavaratti, India; full molecular networking of *M*. *bouillonii* collected from six different locations; ^1^H NMR, ^13^C NMR, COSY, HSQC, HMBC, and NOESY spectra for kavaratamide A (**1**); UV, IR, HRMS, and LC-MS/MS spectra of kavaratamide A (**1**); chiral-phase column chromatography, advanced Marfey’s analysis, and modified Mosher’s ester analysis for kavaratamide A (**1**); MS/MS spectra of kavaratamide A analogues; antitrypanosomal activities and cytotoxicity evaluation in the D283-med cell line (PDF)
