# Supplementary material for: The Kavaratamides: Discovery of Linear Lipodepsipeptides from the Marine Cyanobacterium Moorena bouillonii Using a Comparative Chemogeographic Analysis
Source: J Nat Prod. 2024 Jun 4;87(6):1601–10. doi: 10.1021/acs.jnatprod.4c00242 (PMC11217931; doi:10.1021/acs.jnatprod.4c00242)
Supplement: Supplementary file 1 — np4c00242_si_001.pdf [file np4c00242_si_001.pdf]

Supporting Information

The Kavaratamides: Discovery of Linear Lipodepsipeptides from the Marine Cyanobacterium *Moorena bouillonii* using a Comparative Chemogeographic Analysis

Byeol Ryu,[†] Evgenia Glukhov,[†] Thaiz R. Teixeira,[‡] Conor R. Caffrey,[‡] Saranya Madiyan,[§] Valsamma Joseph,[§] Nicole E. Avalon,[†] Christopher A. Leber,[†] C. Benjamin Naman,[†,⊥] and William H. Gerwick[*, †,∥]*

[†]Center for Marine Biotechnology and Biomedicine, Scripps Institution of Oceanography, University of California San Diego, 9500 Gilman Drive, La Jolla, California 92093, United States.

[‡]Center for Discovery and Innovation in Parasitic Diseases, Skaggs School of Pharmacy and Pharmaceutical Sciences, University of California San Diego, 9500 Gilman Drive, La Jolla, California 92093, United States.

[§]National Centre for Aquatic Animal Health, Cochin University of Science and Technology, Kochi, Kerala 682016, India

[⊥]Department of Science and Conservation, San Diego Botanic Garden, 300 Quail Gardens Drive, Encinitas, California 92024, United States.

[‖]Skaggs School of Pharmacy and Pharmaceutical Sciences, University of California San Diego, 9500 Gilman Drive, La Jolla, California 92093, United States.

Corresponding Author: W. H. Gerwick, email: wgerwick@health.ucsd.edu

Table of Contents

Methods S1. *In vitro* Anti-trypanosomal assay 4

Methods S2. Enzymatic assays of *T. brucei* Cathepsin L-like (*Tbr*CATL) and *T. cruzi* Cruzain (CRZ) proteases 5

Table S1. *M. bouillonii* crude extract sample information 6

Table S2. Antitrypanosomal activity screening results of kavaratamide A (**1**) 7

Figure S1. Map of collection sites of *M. bouillonii* 8

Figure S2. Molecular network of 15 *M. bouillonii* extracts collected from six different locations. 9

Figure S3. LR-LCMS trace of four different *M. bouillonii* extracts from India. 10

Figure S4. DAD trace of four different *M. bouillonii* extracts from India 11

Figure S5. (+) HRESIMS spectrum of kavaratamide A (**1**) 12

Figure S6. UV spectrum of kavaratamide A (**1**) 13

Figure S7. IR spectrum of kavaratamide A (**1**) (ATR) 14

Figure S8. MS/MS fragmentation of kavaratamide A (**1**) 15

Figure S9. Scheme for absolute configuration determination of kavaratmide A (**1**) 16

Figure S10. Modified Mosher's ester analysis of kavaratamide A (**1**) 17

Figure S11. (A) Chiral phase chromatographic analysis for Hiva and (B) HPLC chromatogram of advance Marfey's analysis for Val-1, Val-2, and *N*-Me-Ala. 18

Figure S12. ^{1}H NMR spectrum of kavaratamide A (**1**) in $CDCl_3$ (500 MHz) 19

Figure S13. ^{13}C NMR spectrum of kavaratamide A (**1**) in $CDCl_3$ (125 MHz) 20

Figure S14. COSY spectrum of kavaratamide A (**1**) in $CDCl_3$ (500 MHz) 21

Figure S15. HSQC spectrum of kavaratamide A (**1**) in $CDCl_3$ (600 MHz) 22

Figure S16. HMBC spectrum of kavaratamide A (**1**) in $CDCl_3$ (500 MHz) 23

Figure S17. NOESY spectrum of kavaratamide A (**1**) in $CDCl_3$ (500 MHz) 24

Figure S18. MS/MS spectra of kavaratamides A−C (**1−3**) 25

Figure S19. MS/MS spectra of nodes 4−6 26

Figure S20. Kavaratamides MS/MS-based cluster with the sums of the intensities of the precursor ions of each compound used to determine their relative abundance. These sums were used to depict the node sizes. 27

Figure S21. Concentration response curves of cell viability for D283-med cells treated with kavaratamide A (**1**) (top) and positive control quisinostat (bottom). 28

References 29

Methods S1. ***In Vitro*** **Anti-trypanosomal Assay**

Trypanosoma brucei brucei Lister 427 was cultured at 37 °C under a humidified 5% CO_2 atmosphere in HMI-9 modified medium (Hirumi and Hirumi 1989) supplemented with 20% heat-inactivated fetal bovine serum (FBS). Kavaratamide A (**1**) was diluted in DMSO and added to 96-well polystyrene assay plates to give a final assay concentration of 4 µM (1 µL; 0.5% total DMSO). Fresh HMI-9 medium (99 µL/well) was added to the assay plate. Parasites in the exponential phase were suspended at 2×10^5 parasites/mL in HMI-9 medium and added to each well (100 µL) to a total density of 2×10^4 trypanosomes/well. Assay plates were incubated at 37 °C and 5% CO_2 for 70 h, followed by the addition of 20 µL/well of resazurin 0.5 mM (Faria et al., 2015). The plates were incubated for an additional 2h and fluorescence was measured at 535 nm and 590 nm excitation and emission wavelengths, respectively, using a 2104 EnVision® multilabel plate reader (PerkinElmer, Waltham, MA). The viability of the parasites was normalized to positive and negative controls in each assay plate. The screening was performed in technical quadruplicate, and pentamidine at a fixed concentration of 4 µM was employed as a positive drug control.

Methods S2. Enzymatic assays of *T. brucei* cathepsin L (*Tbr*CATL) and *T. cruzi* cruzain (CRZ)

The recombinant forms of *Tbr*CATL and cruzain were expressed and purified as previously described by Caffrey et al. (2001) and Silva et al. (2019), respectively. Proteolytic activity was measured by monitoring the cleavage of the fluorogenic substrate Z-Phe-Arg-aminomethylcoumarin (Z-FRAMC), in a Synergy HTX (Biotek) fluorimeter. All assays were performed in a 384-well black plate format, at a final volume of 30 µL, in a buffer solution of 0.1 M sodium acetate, pH 5.5, containing 1 mM dithiothreitol, 0.01% Triton X-100, 0.5 nM enzyme, and 2.5 µM substrate. The assay was performed after a 10 min pre-incubation of 10 µM kavaratamide A (**1**) with the enzymes. Initial rates of substrate hydrolysis were calculated relative to a DMSO control. For each assay, two independent experiments were performed, each in triplicate. Trans-Epoxysuccinyl-L-leucylamido(4-guanidino)butane (E-64), at 1 µM, was used as an inhibitor control.

Table S1. *M. bouillonii* crude extract sample information (Leber et al., 2020).

Collection Country /Territory	Collection Region	Alias	Collection code	Collection site	Collection date	Filename
India /Lakshadweep	Kavaratti	India_KP	KP-16	Paradise Hut lagoon	2/6/2016	2019-08-23_ CBN_KP-16-1
India /Lakshadweep	Kavaratti	India_KSP	KSP07APR18-1	south of Paradise Hut pier	4/7/2018	2019-08-23_ CBN_KSP-18-1
India /Lakshadweep	Kavaratti	India_KPL	KPL08APR18-1	Paradise Hut lagoon	4/8/2018	2019-08-23_ CBN_KPL-18-1
India /Lakshadweep	Kavaratti	India_KHI	KHT08APR18-3	Heaven's Treat lagoon	4/8/2018	2019-08-23_ CBN_KHI-18-1
China /Xisha	Sanshax	China_13	XSSCB2017_13	16° 51' 05.52", 112° 20' 56.13"	5/16/2017	20170915_ CBN_XSSCB2017_13
China /Xisha	Sanshax	China_24	XSSCB2017_24	16° 51' 05.52", 112° 20' 56.13"	5/19/2017	20170915_ CBN_XSSCB2017_24
China /Xisha	Sanshax	China_25	XSSCB2017_25	16° 51' 05.52", 112° 20' 56.13"	5/19/2017	20170915_ CBN_XSSCB2017_25
Saipan	-	Saipan_00	SPB31JAN13-1	Laulau Bay	1/31/2013	2200
Saipan	-	Saipan_09	SPD29JAN13-6	Laulau Bay	1/29/2013	2209
Saipan	-	Saipan_32	SPB01FEB13-1	Laulau Bay	2/1/2013	2232
American Samoa	-	AmSam_20	ASA12JUL14-1	Afao	7/12/2014	2220
American Samoa	-	AmSam_23	ASG15JUL14-1	Fagasa Bay	7/15/2014	2223
Guam	-	Guam_46	GBB21MAR16-1	Apra Harbor	3/21/2016	2246
Guam	-	Guam_47	GGG21MAR16-1	Apra Harbor	3/21/2016	2247
Papua New Guinea	New Ireland	PNG_c	PNG19MAY05-8	Pigeon Island	5/19/2005	Mb

Table S2. Antitrypanosomal activity screening results of kavaratamide A (**1**)

**Compound**	**% inhibition *T. b. brucei* (4 µM)**	**% inhibition *Tbr*CATL (10 µM)**	**% inhibition cruzain (10 µM)**
Kavaratamide A (**1**)	28 ± 5	0 ± 3	0 ± 2
Pentamidine*	100 ± 1	NA	NA
E-64 (1 µM)**	NA	99 ± 0	97 ± 2

NA: not applicable. *Positive control for *T. b. brucei* assay; ** Positive control for enzymatic assay.

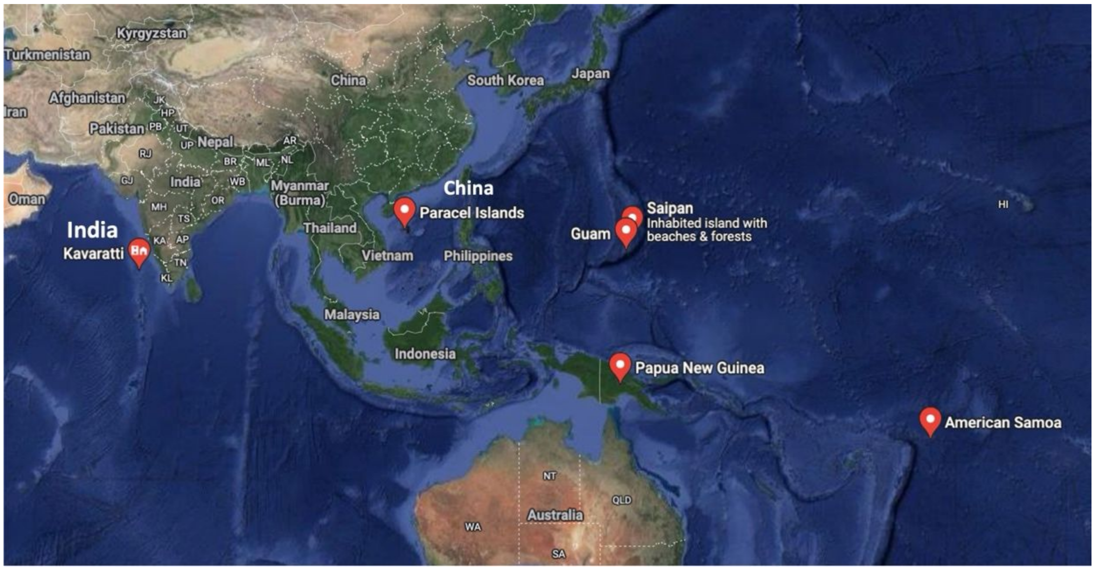

Figure S1. Map of collection sites of *M. bouillonii*

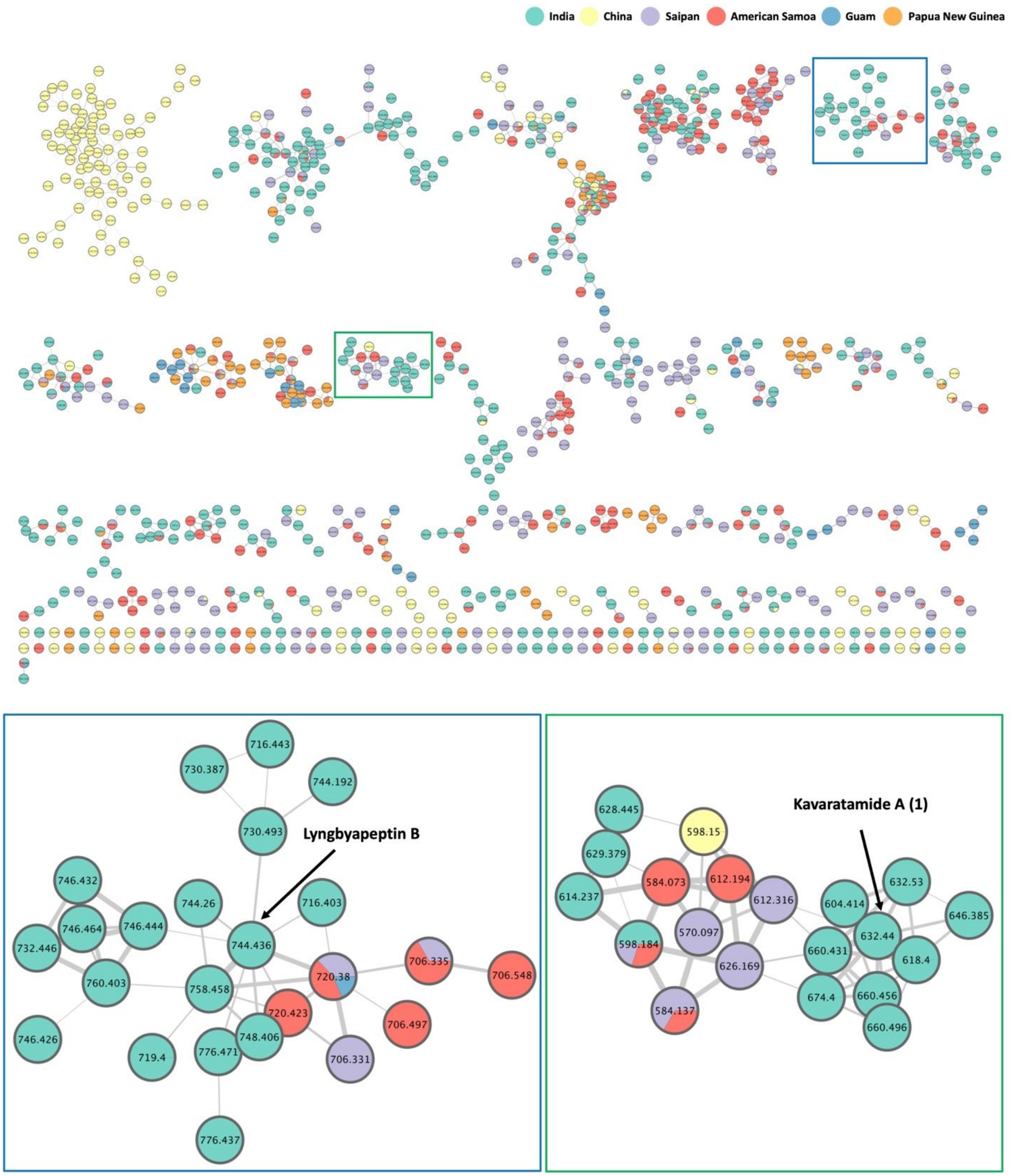

Figure S2. Molecular network of 15 *M. bouillonii* extracts collected from six different locations. The clusters possessing at least two nodes appear in this figure.

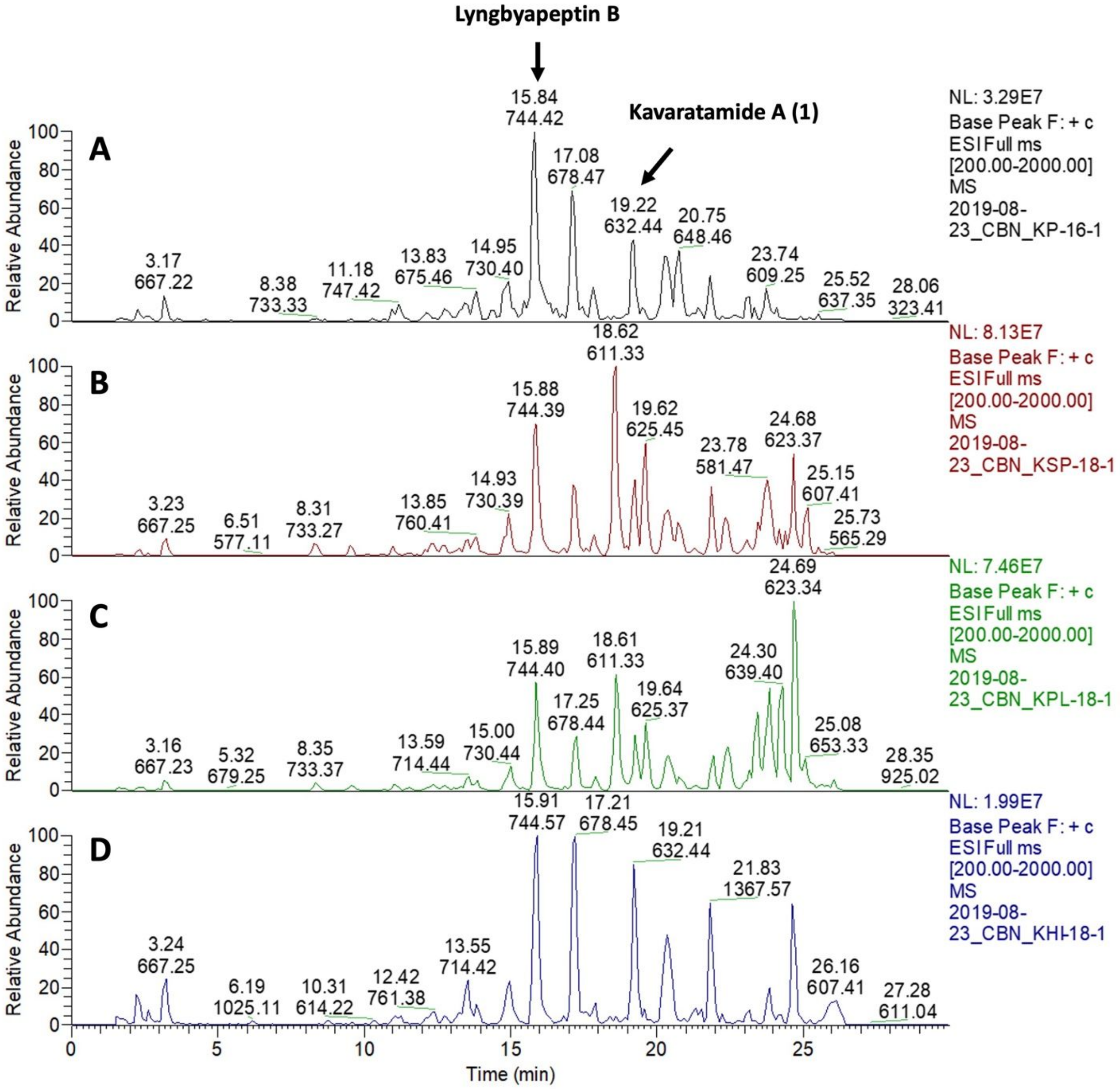

Figure S3. LR-LCMS trace of four different *M. bouillonii* extracts from India. The collection codes are as follow: (A) KP-16, (B) KSP07APR18-1, (C) KPL08APR18-1, and (D) KHT08APR18-3.

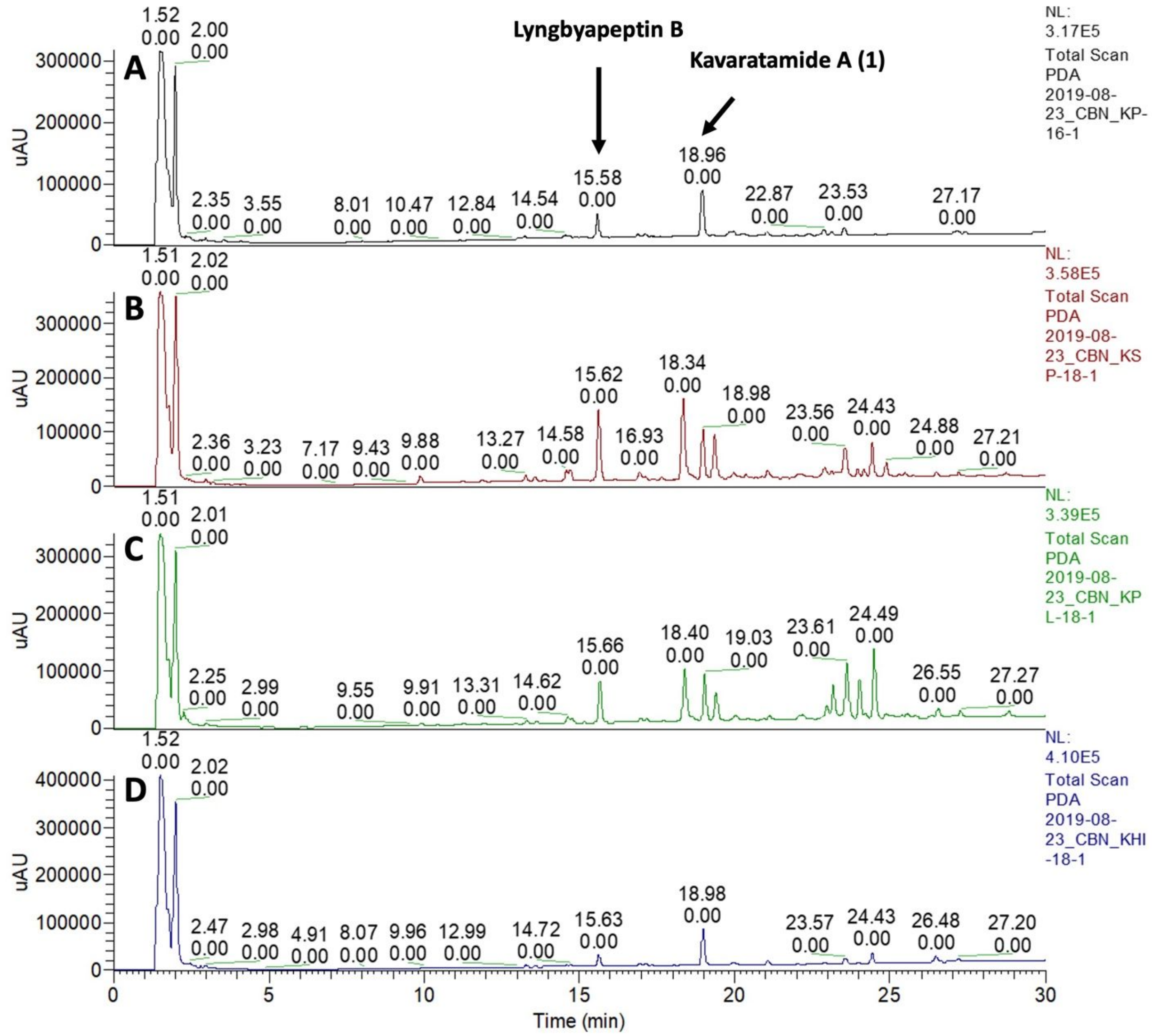

Figure S4. DAD trace of four different *M*. *bouillonii* extracts from India. The collection codes are as follow: (A) KP-16, (B) KSP07APR18-1, (C) KPL08APR18-1, and (D) KHT08APR18-3. The peak at 15.58 min and 18.96 min are lyngbyapeptin B and kavaratamide A (**1**), respectively.

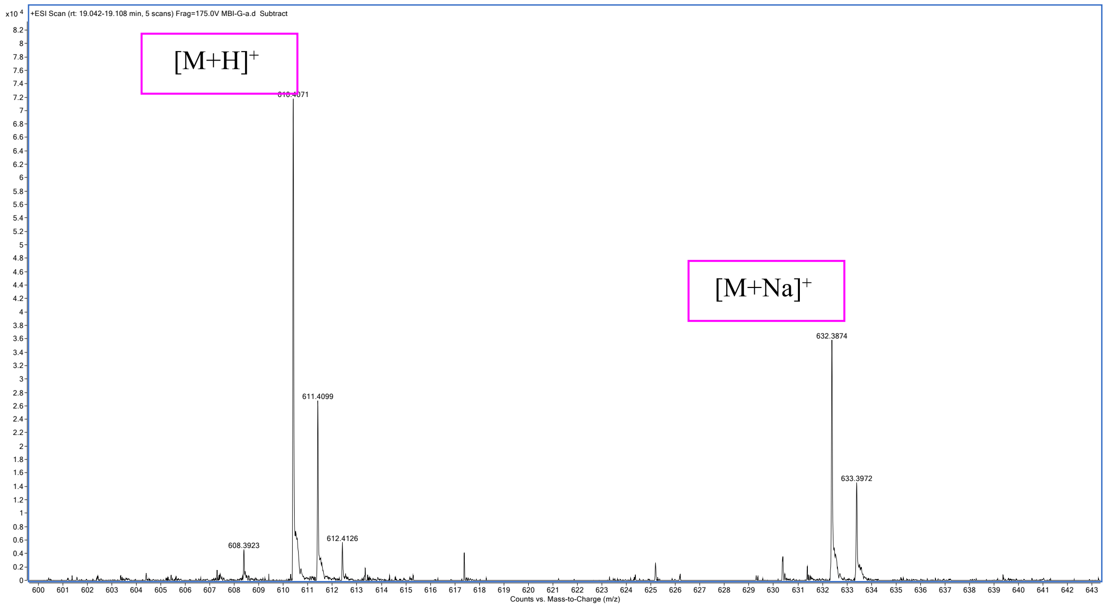

Mass Measured	Theo. Mass	Delta (ppm)	Composition
610.4071	610.4062	1.5	$[C_{32}H_{56}N_3O_8]^+$
632.3874	632.3881	-1.1	$[C_{32}H_{55}N_3O_8Na]^+$

Figure S5. HRESI(+)MS spectrum of kavaratamide A (**1**).

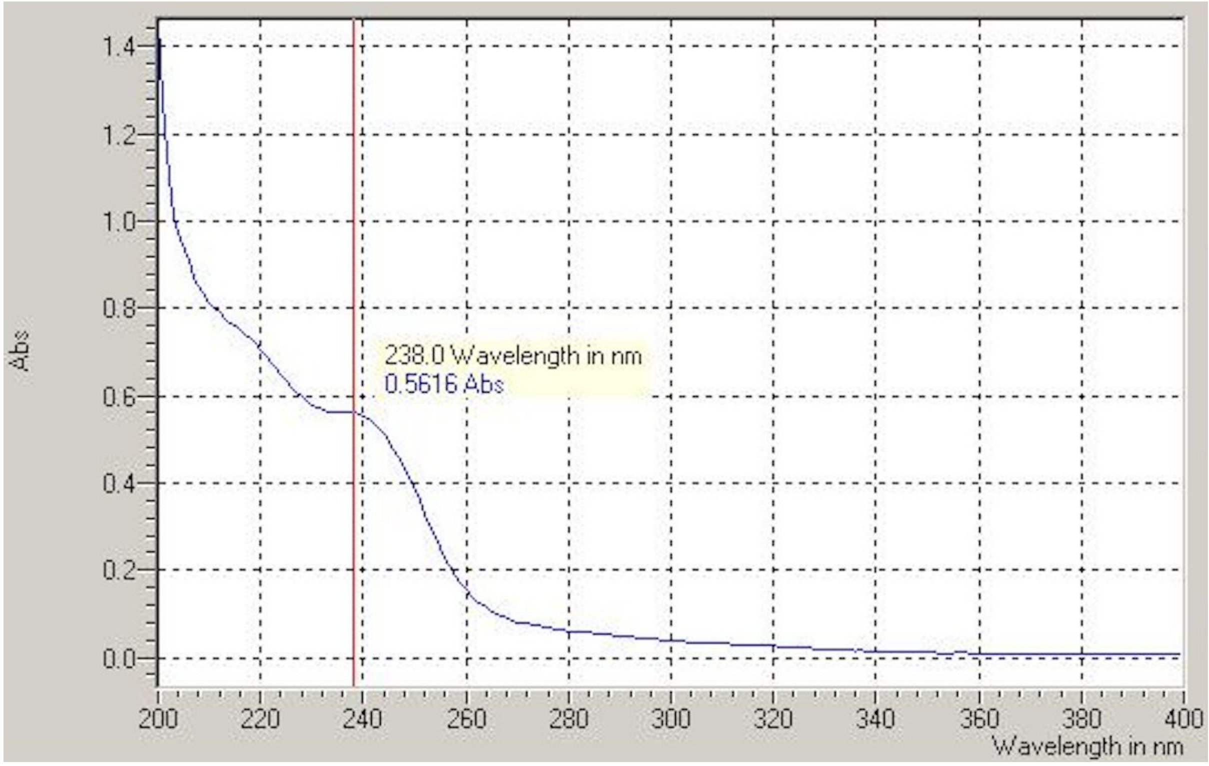

Figure S6. UV spectrum of kavaratamide A (**1**).

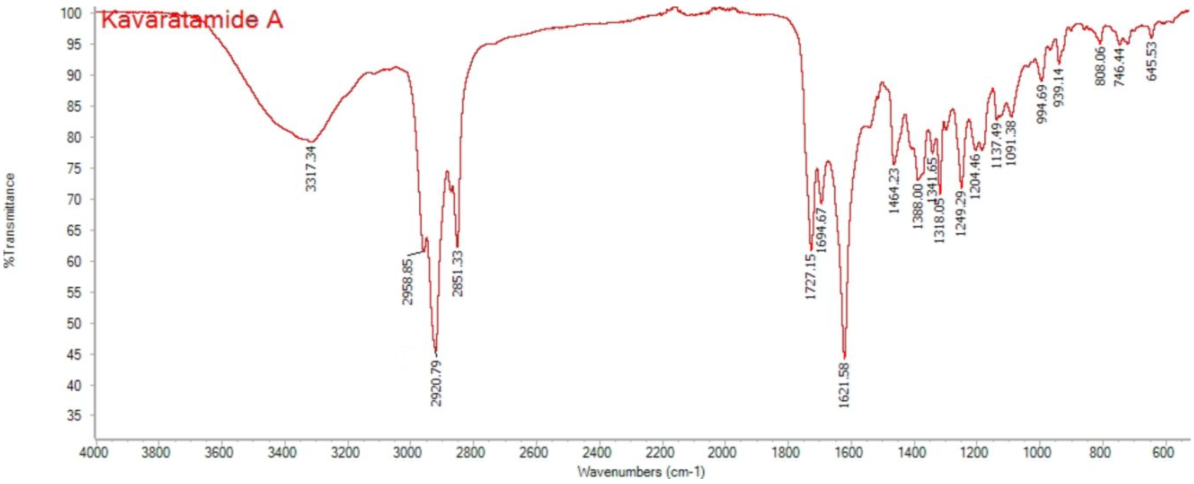

Figure S7. IR spectrum of kavaratamide A (**1**) (ATR).

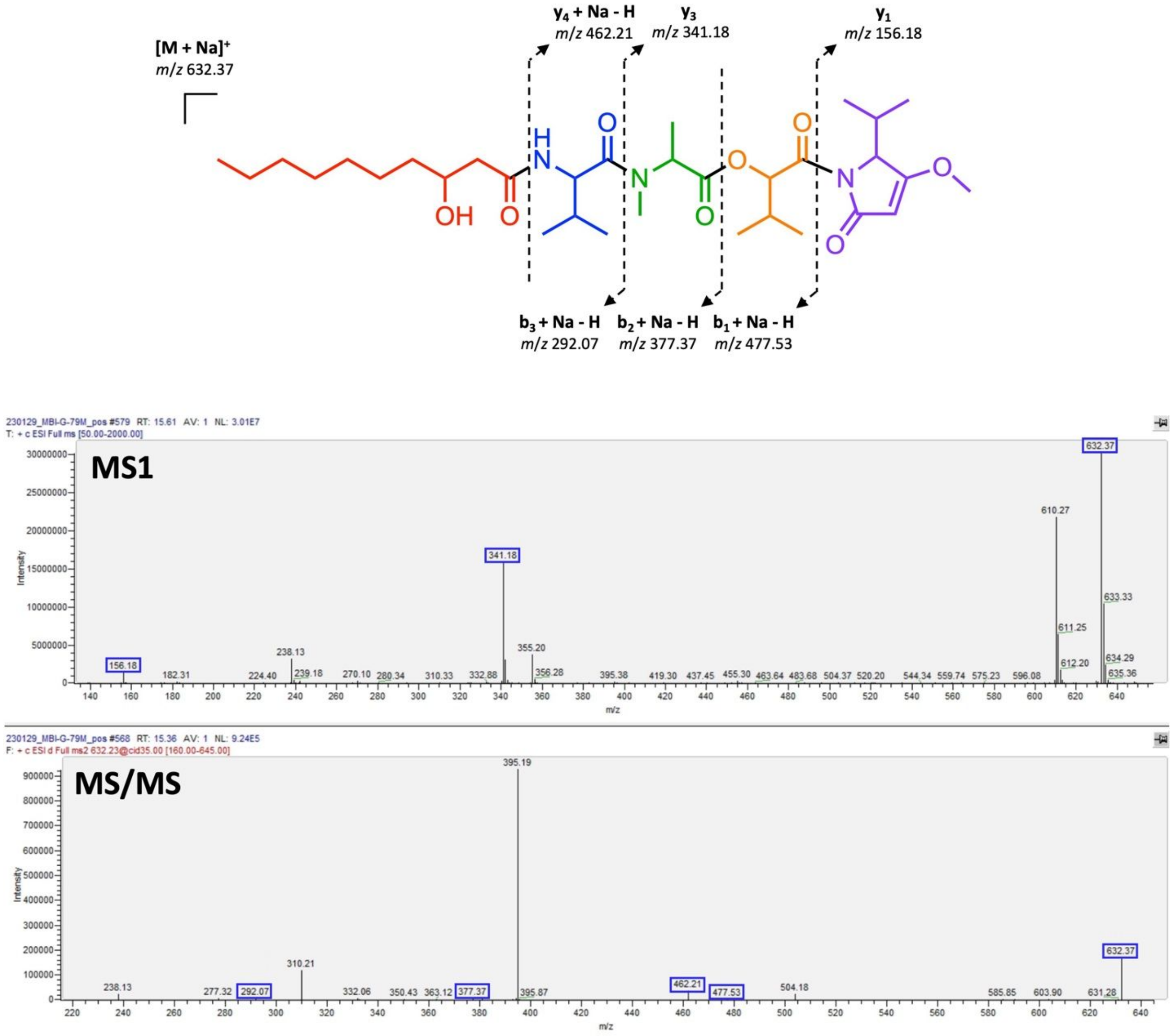

Figure S8. MS/MS fragmentation of kavaratamide A (**1**).

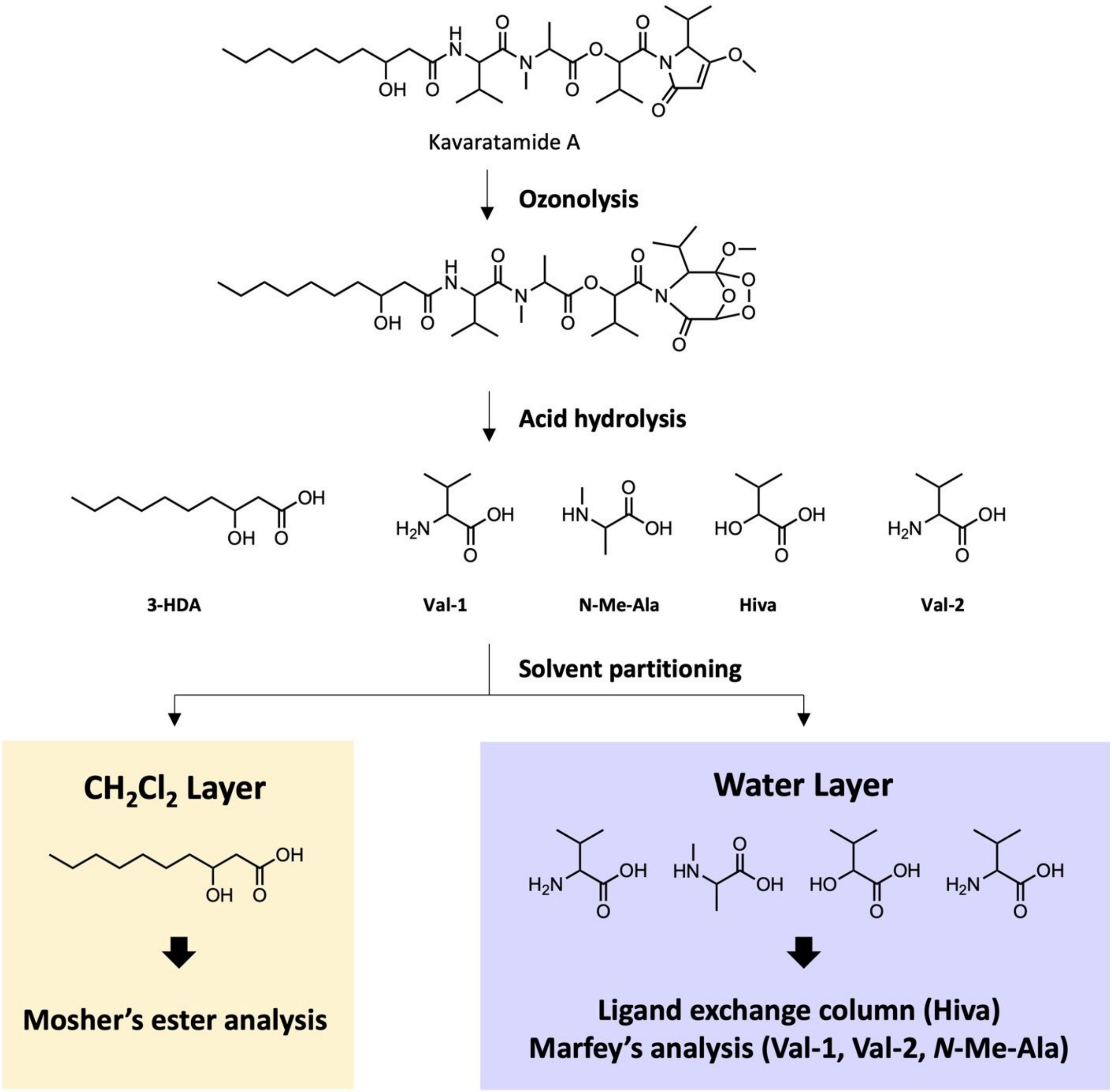

Figure S9. Scheme for absolute configuration determination of kavaratamide A (**1**).

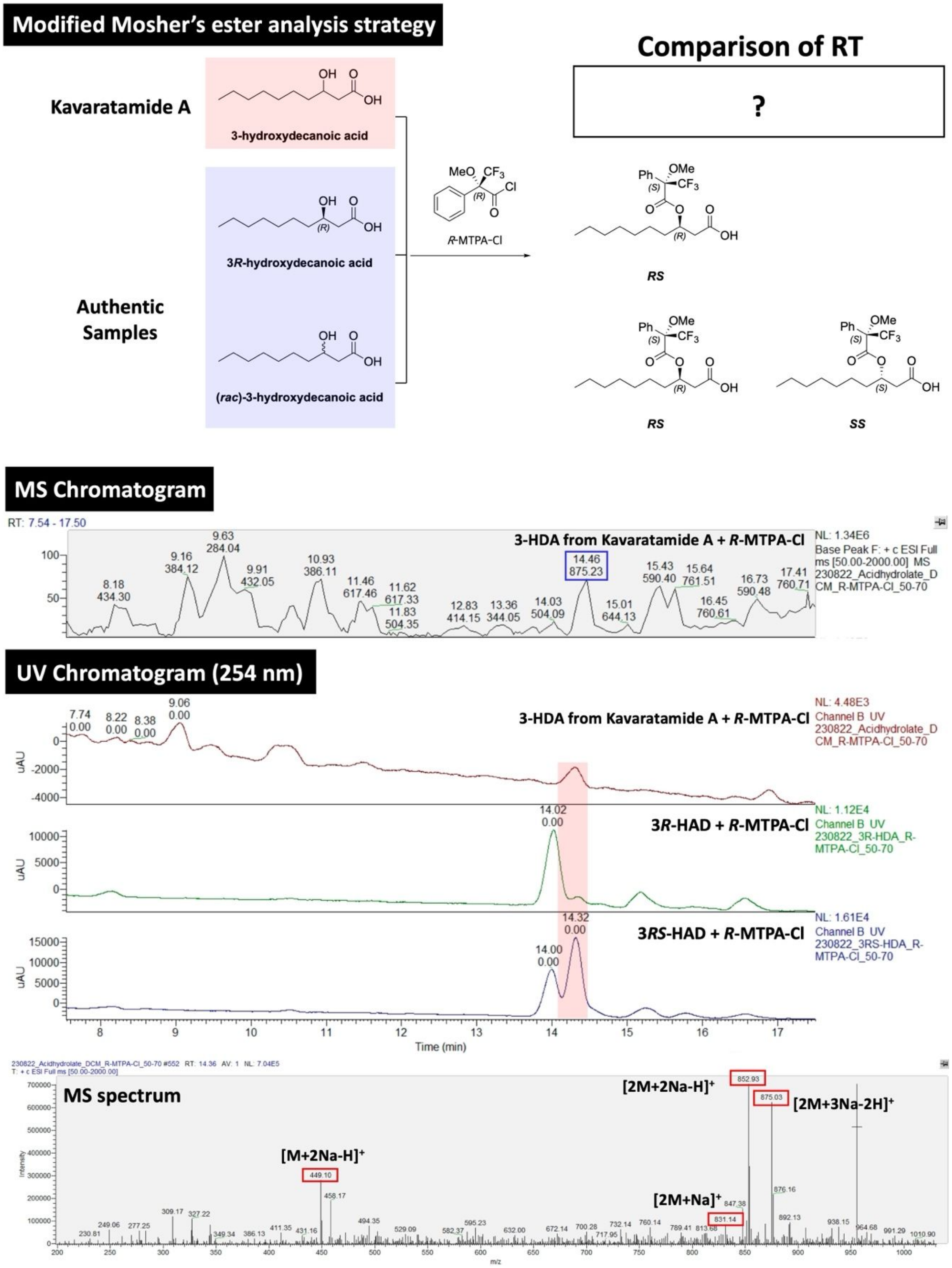

Figure S10. Modified Mosher's ester analysis of kavaratamide A (**1**).

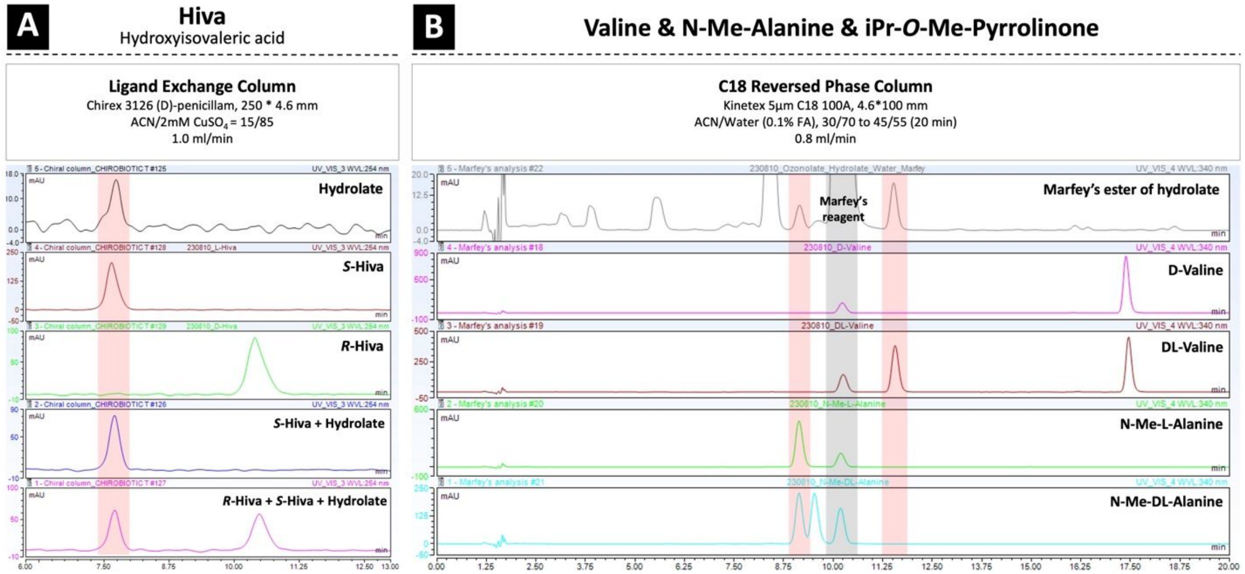

Figure S11. (A) Chiral phase chromatographic analysis for Hiva and (B) HPLC chromatogram of advance Marfey's analysis for Val-1, Val-2, and *N*-Me-Ala.

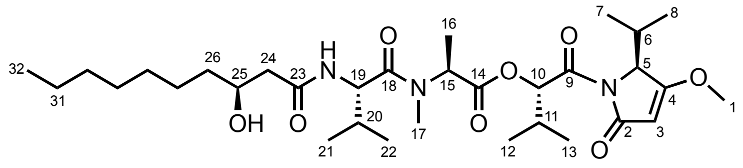

Kavaratamide A (**1**)

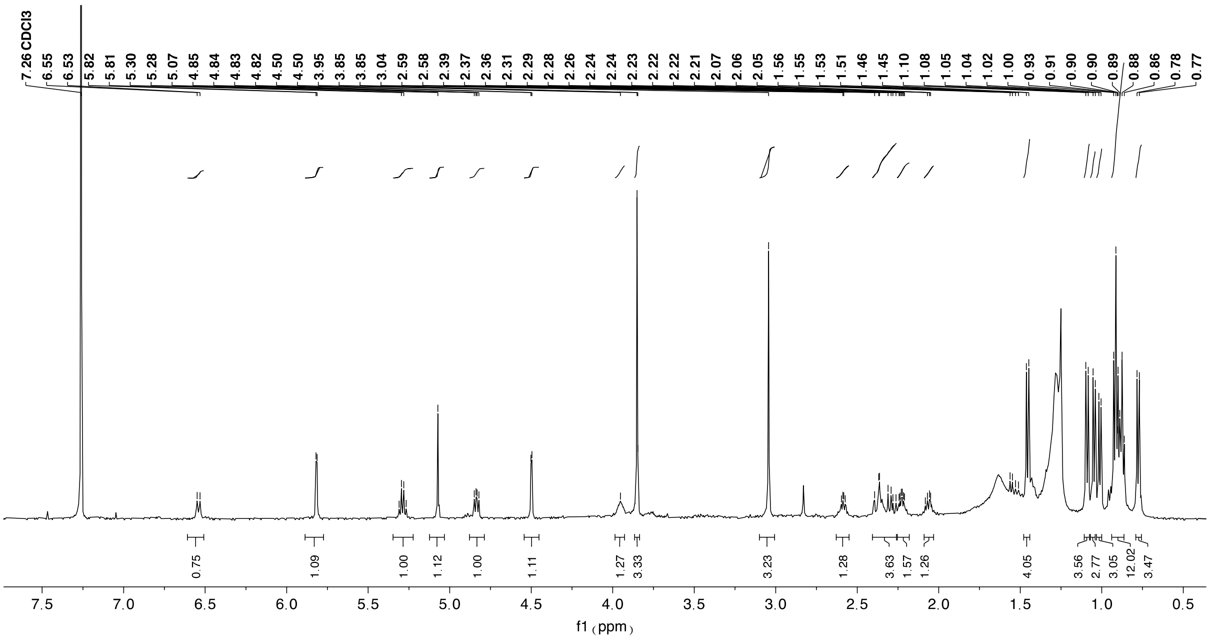

Figure S12. ^{1}H NMR spectrum of kavaratamide A (**1**) in $CDCl_3$ (500 MHz).

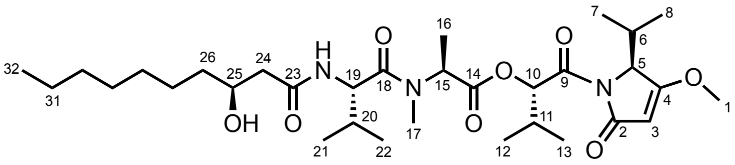

Kavaratamide A (**1**)

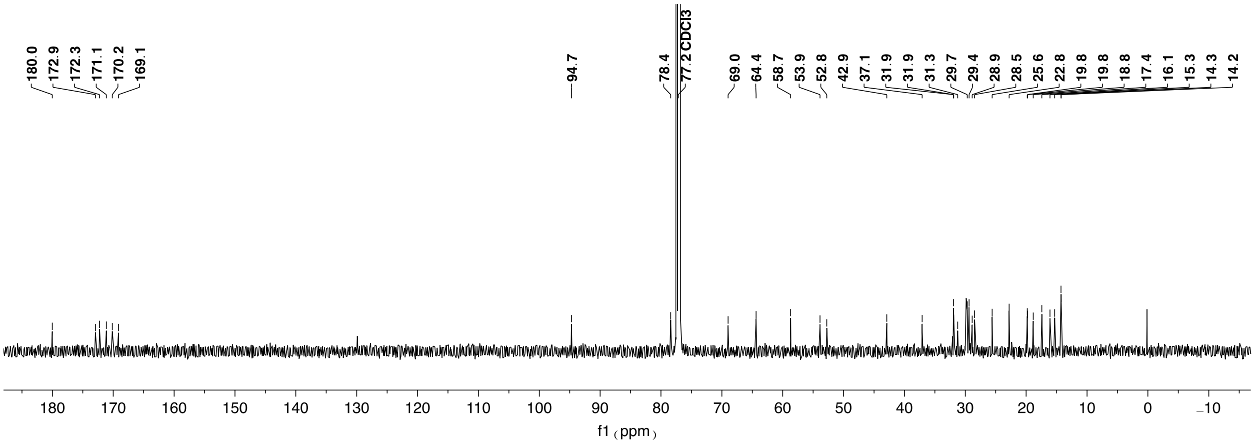

Figure S13. ^{13}C NMR spectrum of kavaratamide A (**1**) in $CDCl_3$ (125 MHz).

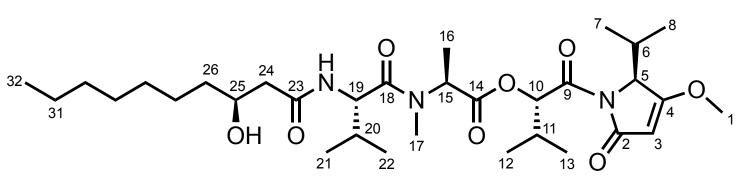

Kavaratamide A (**1**)

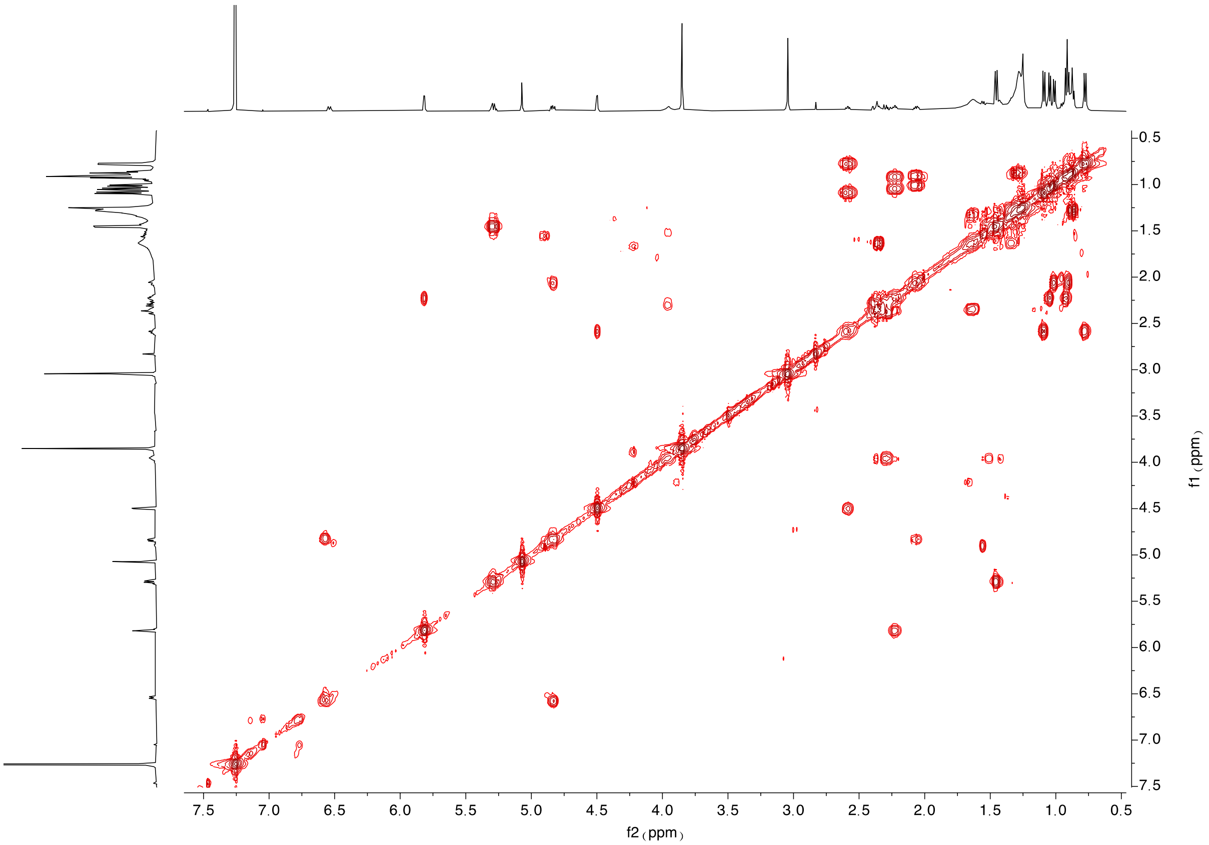

Figure S14. COSY spectrum of kavaratamide A (**1**) in $CDCl_3$ (500 MHz).

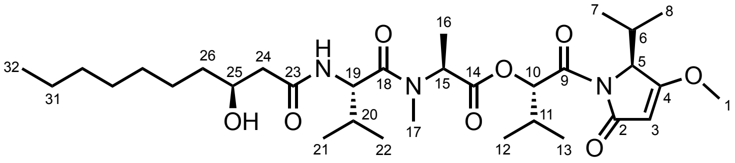

Kavaratamide A (**1**)

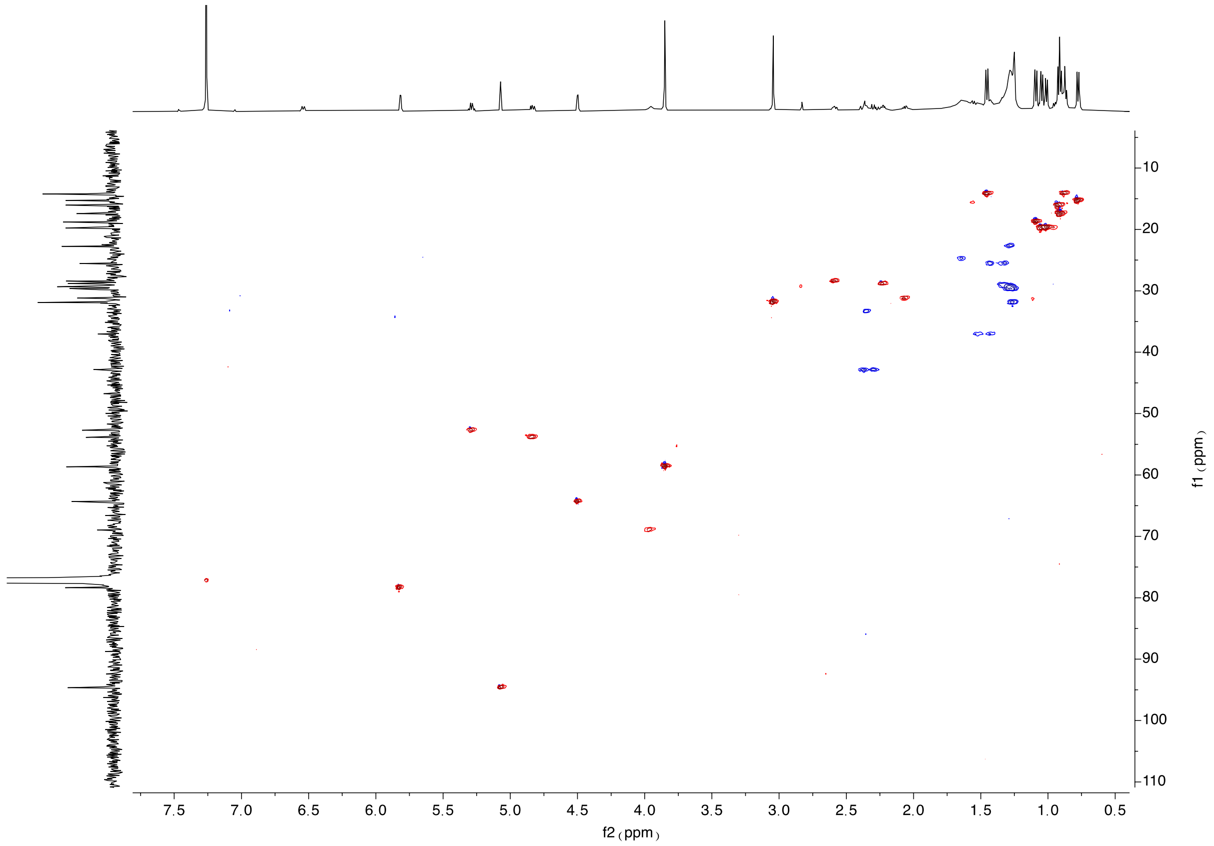

Figure S15. HSQC spectrum of kavaratamide A (**1**) in $CDCl_3$ (600 MHz).

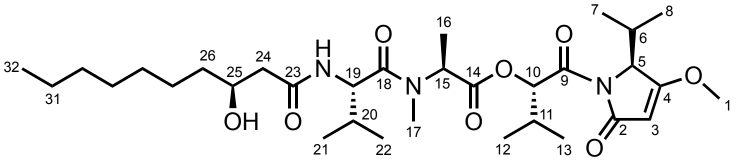

Kavaratamide A (**1**)

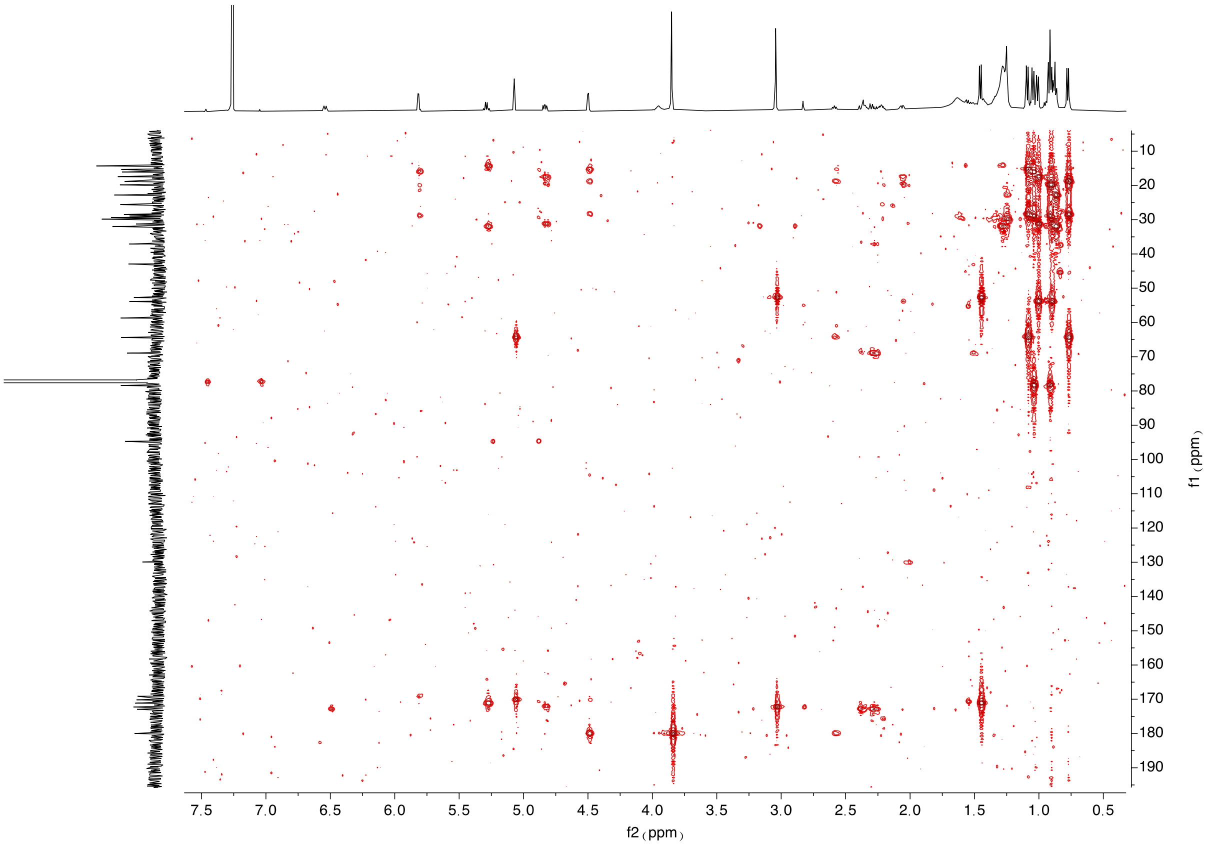

Figure S16. HMBC spectrum of kavaratamide A (**1**) in $CDCl_3$ (500 MHz).

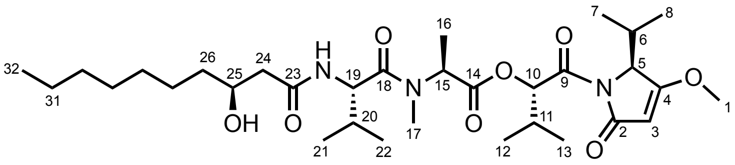

Kavaratamide A (**1**)

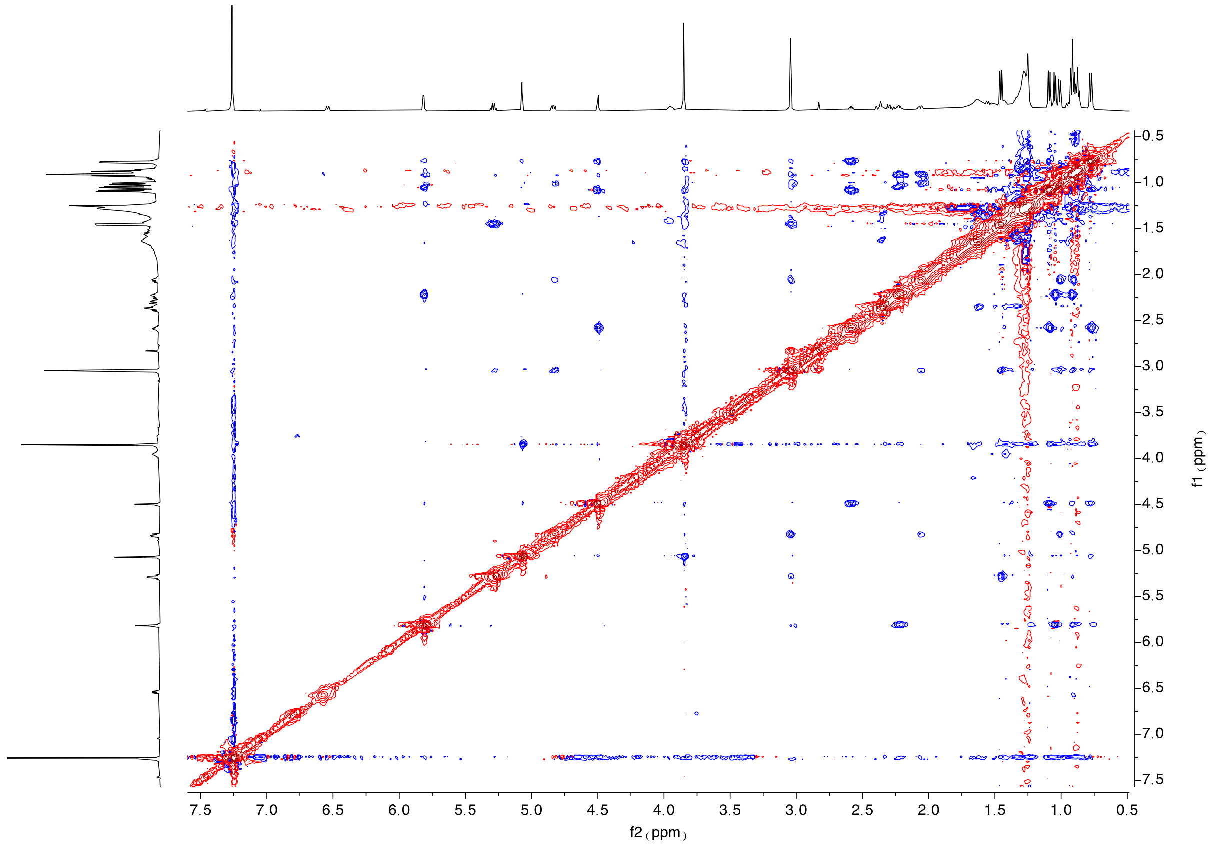

Figure S17. NOESY spectrum of kavaratamide A (**1**) in $CDCl_3$ (500 MHz).

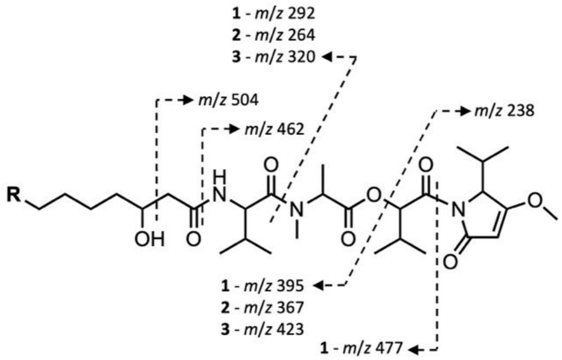

Compound	R	Fatty acid moiety
Kavaratamide A (**1**)	$-(CH_2)_2CH_3$	3-Hydroxydecanoic acid
Kavaratamide B (**2**)	$-CH_3$	3-Hydroxyoctanoic acid
Kavaratamide C (**3**)	$-(CH_2)_4CH_3$	3-Hydroxydodecanoic acid

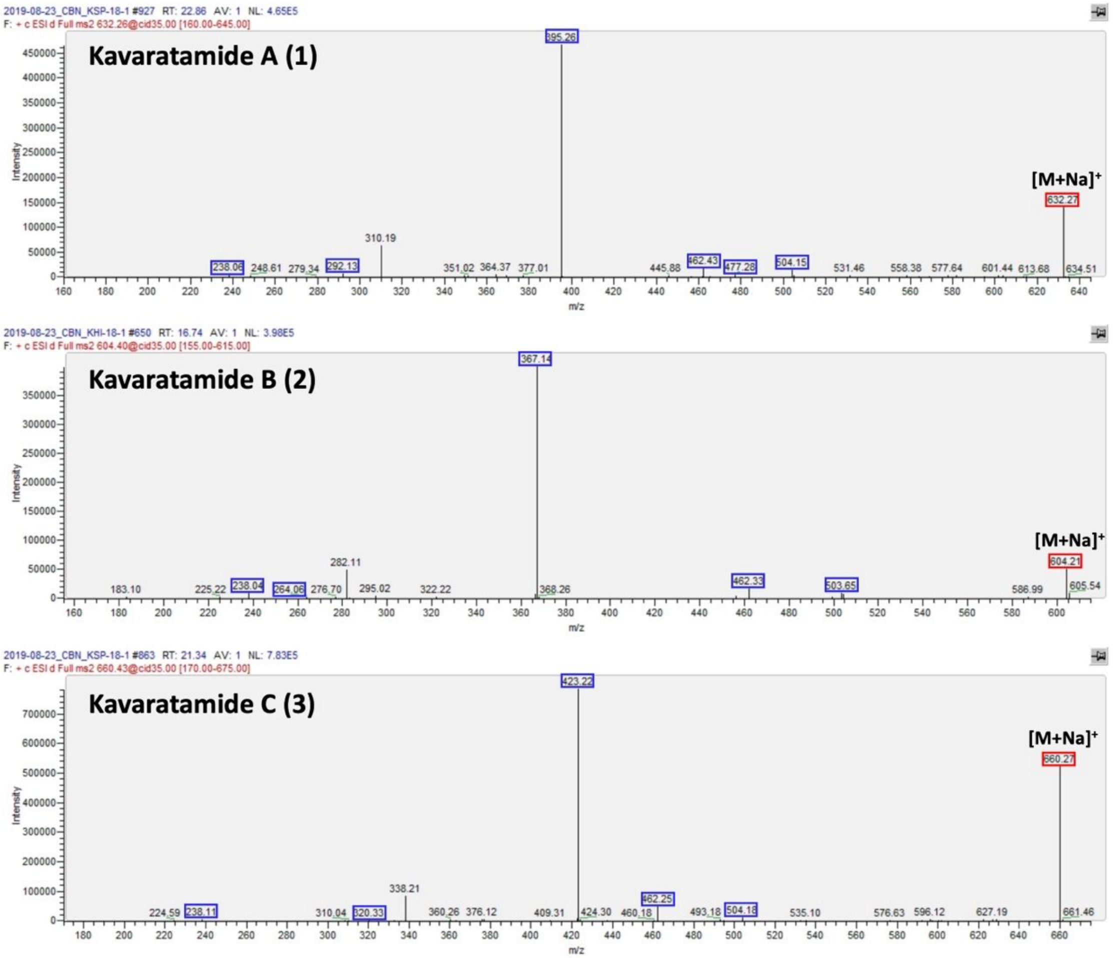

Figure S18. MS/MS spectra of kavaratamides A−C (**1**−**3**).

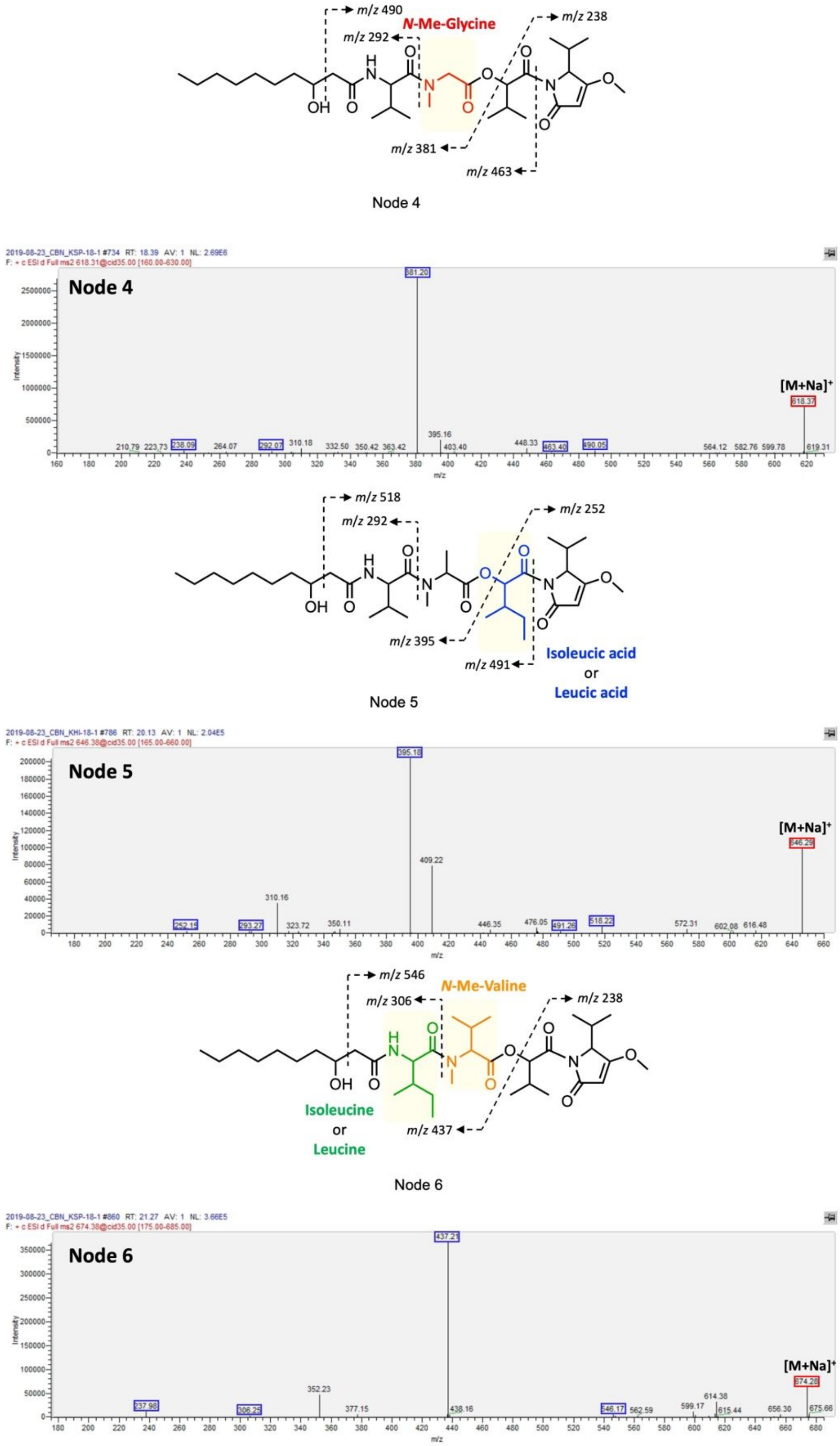

Figure S19. MS/MS spectra of Nodes 4−6.

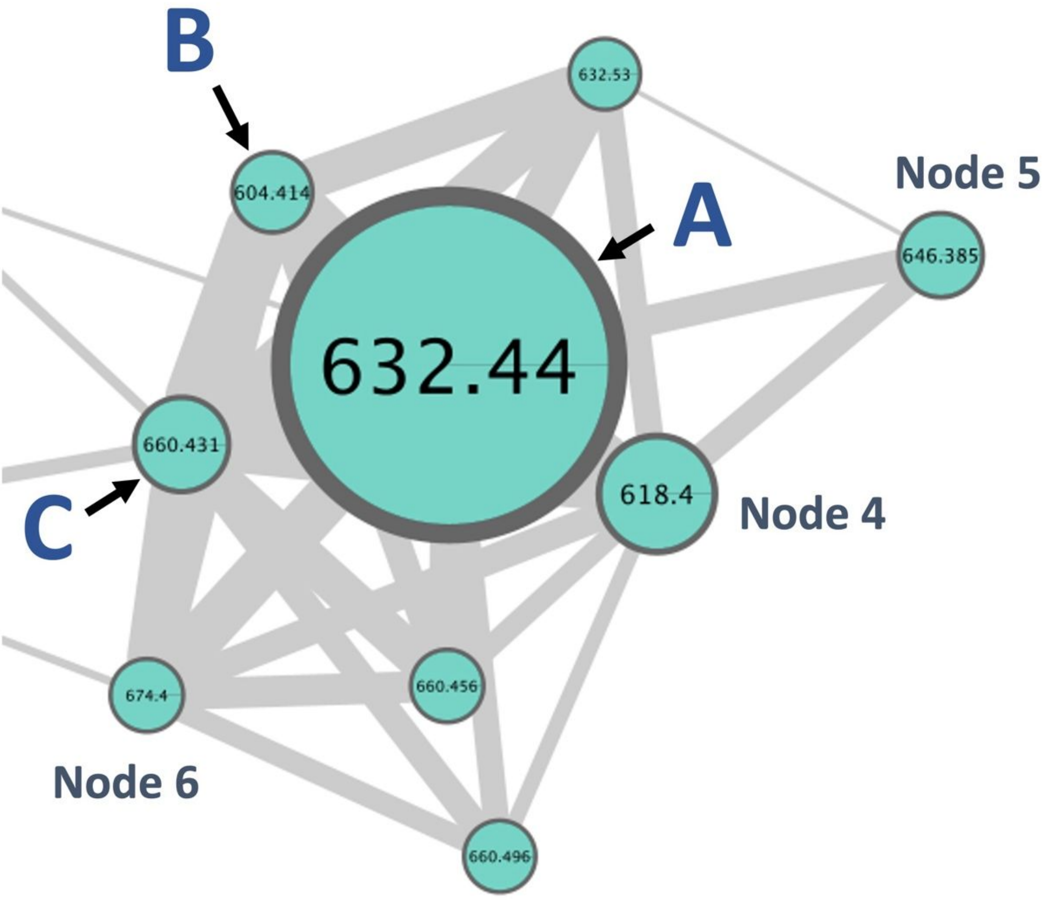

	parent mass	sum(precursor intensity)
Kavaratamide A (**1**) →	632.44	7.87173E7
Node 4 →	618.4	1.34428E7
Kavaratamide C (**3**) →	660.431	7018440.0
Node 5 →	646.385	3984970.0
Kavaratamide B (**2**) →	604.414	2836670.0
Node 6 →	674.4	712262.0
	660.456	657864.0
	660.496	344966.0
	632.53	151167.0

Figure S20. Kavaratamides MS/MS-based cluster with the sums of the intensities of the precursor ions of each compound used to determine their relative abundance. These sums were used to depict the node sizes.

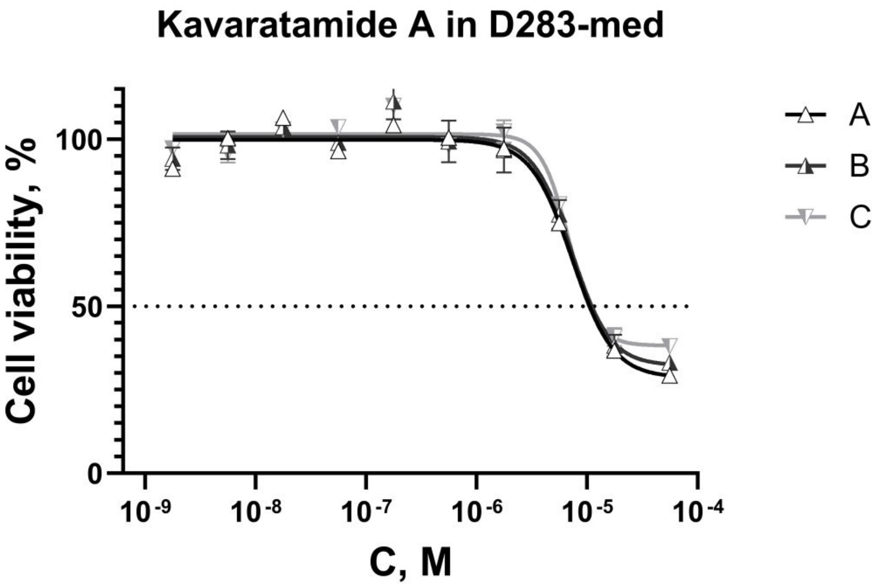

	A	B	C
IC50	7.308e-006	7.233e-006	6.852e-006

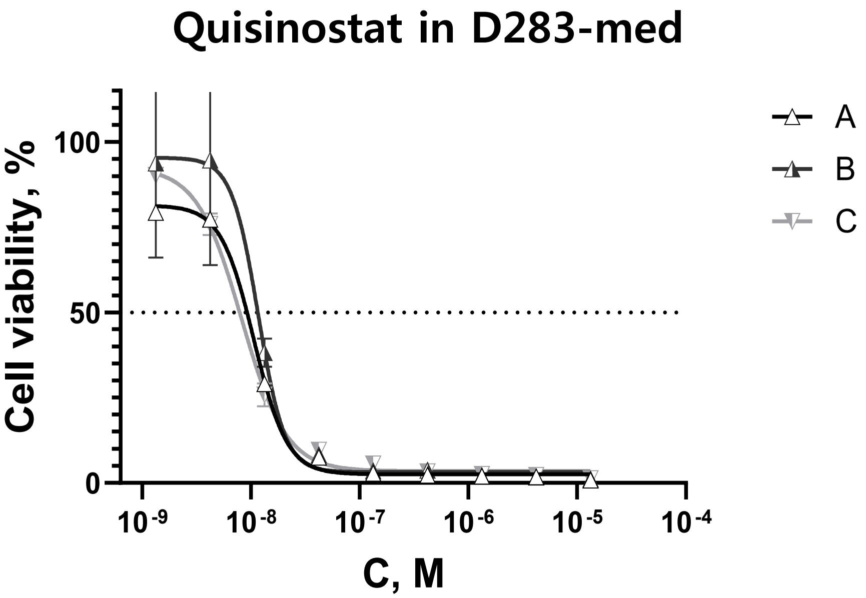

	A	B	C
IC50	1.065e-008	1.167e-008	8.213e-009

Figure S21. Concentration response curves of cell viability for D283-med cells treated with kavaratamide A (**1**)(top) and positive control quisinostat (bottom).

References:

Hirumi, H; Hirumi, K. Continuous cultivation of *Trypanosoma brucei* blood stream forms in a medium containing a low concentration of serum protein without feeder cell layers. J. Parasitol. **1989**, 75, 985-989.

Faria, J; Moraes, CB; Song, R; Pascoalini, BS; Lee, N; Siqueira-Neto, JL; Cruz, DJM; Parkinson, T; Ioset, JR; Cordeiro-da-Silva, A; Freitas-Junior, LH. Drug discovery for human African trypanosomiasis: identification of novel scaffolds by the newly developed HTS SYBR Green assay for *Trypanosoma brucei*. J. Biomol. Screen. **2015**, 20, 70-81.

Silva, EB; Dall, E; Briza, P; Brandstetter, H; Ferreira, RS. Cruzain structures: apocruzain and cruzain bound to S-methyl thiomethanesulfonate and implications for drug design. Acta Crystallogr., Sect. F: Struct. Biol. Commun. **2019**, 75, 419–427.

Caffrey, CR; Hansell, E; Lucas, KD; Brinen, LS; Alvarez Hernandez, A; Cheng, J; Gwaltney, SL; Roush, WR; Stierhof, YD; Bogyo, M; Steverding, D; McKerrow, JH. Active site mapping, biochemical properties and subcellular localization of rhodesain, the major cysteine protease of *Trypanosoma brucei rhodesiense*. Mol. Biochem. Parasitol. **2001**, 118, 61–73.

Leber, C. A.; Naman, C. B.; Keller, L.; Almaliti, J.; Caro-Diaz, E. J. E.; Glukhov, E.; Joseph, V.; Sajeevan, T. P.; Reyes, A. J.; Biggs, J. S. Applying a Chemogeographic Strategy for Natural Product Discovery from the Marine Cyanobacterium *Moorena bouillonii*. *Mar. Drugs* **2020**, *18*, 515.
